# ZmEREB92 plays a negative role in seed germination by regulating ethylene signaling and starch mobilization in maize

**DOI:** 10.1371/journal.pgen.1011052

**Published:** 2023-11-17

**Authors:** Jingye Fu, Wenzheng Pei, Linqian He, Ben Ma, Chen Tang, Li Zhu, Liping Wang, Yuanyuan Zhong, Gang Chen, Qi Wang, Qiang Wang

**Affiliations:** 1 State Key Laboratory of Crop Gene Exploration and Utilization in Southwest China, College of Agronomy, Sichuan Agricultural University, Chengdu, China; 2 Graduate School of Horticulture, Chiba University, Matsudo, Chiba, Japan; 3 Key Laboratory of Aquatic Genomics, Ministry of Agriculture and Rural Affairs, Beijing Key Laboratory of Fishery Biotechnology, Chinese Academy of Fishery Sciences, Beijing, China; Max Planck Institute of Molecular Plant Physiology: Max-Planck-Institut fur molekulare Pflanzenphysiologie, GERMANY

## Abstract

Rapid and uniform seed germination is required for modern cropping system. Thus, it is important to optimize germination performance through breeding strategies in maize, in which identification for key regulators is needed. Here, we characterized an AP2/ERF transcription factor, ZmEREB92, as a negative regulator of seed germination in maize. Enhanced germination in *ereb92* mutants is contributed by elevated ethylene signaling and starch degradation. Consistently, an ethylene signaling gene *ZmEIL7* and an α-amylase gene *ZmAMYa2* are identified as direct targets repressed by ZmEREB92. *OsERF74*, the rice ortholog of *ZmEREB92*, shows conserved function in negatively regulating seed germination in rice. Importantly, this orthologous gene pair is likely experienced convergently selection during maize and rice domestication. Besides, mutation of *ZmEREB92* and *OsERF74* both lead to enhanced germination under cold condition, suggesting their regulation on seed germination might be coupled with temperature sensitivity. Collectively, our findings uncovered the ZmEREB92-mediated regulatory mechanism of seed germination in maize and provide breeding targets for maize and rice to optimize seed germination performance towards changing climates.

## Author summary

Seed germination is a crucial step for agricultural production, yet the regulatory mechanism in many crops remains unclear. We demonstrated that ZmEREB92 negatively regulates maize seed germination in a temperature-sensitive manner. In suitable condition, ZmEREB92 is transcriptionally inhibited during imbibition, thereby releasing the expression of *ZmEIL7* and *ZmAMYa2* to enhance ethylene signaling and starch degradation, which ensures timely germination. When the temperature is low, the expression of *ZmEREB92* is strongly induced after imbibition. The increased level of *ZmEREB92* directly represses the transcription of *ZmEIL7* and *ZmAMYa2* by binding to their promoters, leading to inhibited seed germination. The rice ortholog of *ZmEREB92*, *OsERF74*, is functionally conserved in controlling seed germination in rice, and this orthologous gene pair show evidences of selection during maize and rice domestication.

## Introduction

With the importance as food, feed and bioethanol, maize productivity requires to be improved to deal with the growing population and climate uncertainty [1,2]. Deficiency in seed germination leads to poor seedling establishment, consequently reducing the yield [3,4]. Since the cultivation area of maize expanded from tropics to temperate regions, the germination defects of maize occur more frequently due to the low temperature [5–7]. Thus, identification of key regulators in maize seed germination to optimize germination performance with breeding technologies will be beneficial for maize production.

Seed germination begins with imbibition and ends with radicle emergence, which depends on the expansion of embryo and the rupture of surrounding structures [8]. Like most cereal crops, maize seed possesses a massive starchy endosperm that serves as the principal storage organ, in which hydrolysis of starch by starch-degrading enzymes, particularly α-amylase, provides most growth energy [8–10]. It has been well-established for the central roles of gibberellin (GA) and abscisic acid (ABA) in precisely regulating seed germination, in which GA signaling promotes germination by countering the inhibition executed by ABA [11,12]. Besides, seed germination is also controlled by other phytohormones. For instance, in Arabidopsis, the JAZ proteins, repressors of jasmonic acid (JA) signaling, interacts with ABA-responsive ABI3 to mediate the synergistic inhibition of ABA and JA on seed germination [13]. Rice BZR1 transcription factor directly targets *RAmy3D* to enhance starch mobilization through brassinosteroid signaling during germination [14]. Ethylene, the gaseous phytohormone, also promotes seed germination, which involves the antagonistic effect with ABA signaling to boost dormancy release [12,15]. Ethylene insensitive mutants (*etr1*, *ein2*, and *ein6*) are hypersensitive to ABA with enhanced seed dormancy, whereas *eto1*, *eto3*, and *ctr1* mutants, which showed increased ethylene production, are desensitized to ABA and germinated stronger [16–18]. The APETALA2/Ethylene Responsive Factors (AP2/ERFs) play pivotal roles in regulating hormone-mediated seed germination. AtABI4 was found to up-regulate ABA catabolism genes but down-regulate GA biosynthesis genes in Arabidopsis, thereby leading to increased seed germination in *abi4* mutant [19,20]. In rice, OsAP2-39 negatively regulates seed germination by directly targeting to ABA biosynthesis gene *OsNCED9* and GA-inactivating gene *OsEUI* [21]. Nevertheless, the role of AP2/ERF transcription factors controlling seed germination in maize remains elusive.

Transcriptomics demonstrated a conspicuous enrichment of ethylene-related genes in addition to ABA/GA-related genes during seed germination in maize [22,23]. Moreover, quantitative trait loci (QTLs) controlling ethylene production were identified in a maize recombinant inbred line (RIL) mapping population constructed from two parental lines with different germination capability [24]. More recently, by genome-wide association study (GWAS) and transcriptomics, an ethylene-responsive transcription factor ZmMADS26 was identified to regulate seed germination in maize [25]. These findings strongly imply the action of ethylene in regulating maize seed germination.

With similar demands for synchronous germination in modern crop cultivation, seed dormancy and germination turns to be the traits that have experienced convergently selection during crop domestication [26,27]. The *Seed dormancy 4* (*Sdr4*) is the first domesticated gene identified in rice that controls seed dormancy [28]. The *G* gene possess conserved function in controlling seed dormancy and showed evidence of parallel selection during domestication in soybean, rice and tomato [29]. Here, we identified ZmEREB92 as a negative regulator in maize seed germination by directly repressing the transcription of an ethylene signaling gene, *ZmEIL7* and an α-amylase gene *ZmAMYa2*, thereby leading to enhanced embryo growth with elevated ethylene signaling and endosperm starch mobilization in *ereb92* mutants. A rice ortholog of *ZmEREB92* is identified as *OsERF74*, which shows conserved function in regulating rice seed germination. Both *ZmEREB92* and *OsERF74* might have undergone selection during domestication. Moreover, this orthologous gene pair also possess the capability to regulate seed germination under cold stress.

## Results

### ZmEREB92 negatively regulates seed germination in maize by affecting embryo growth during imbibition

**Fig 1:**
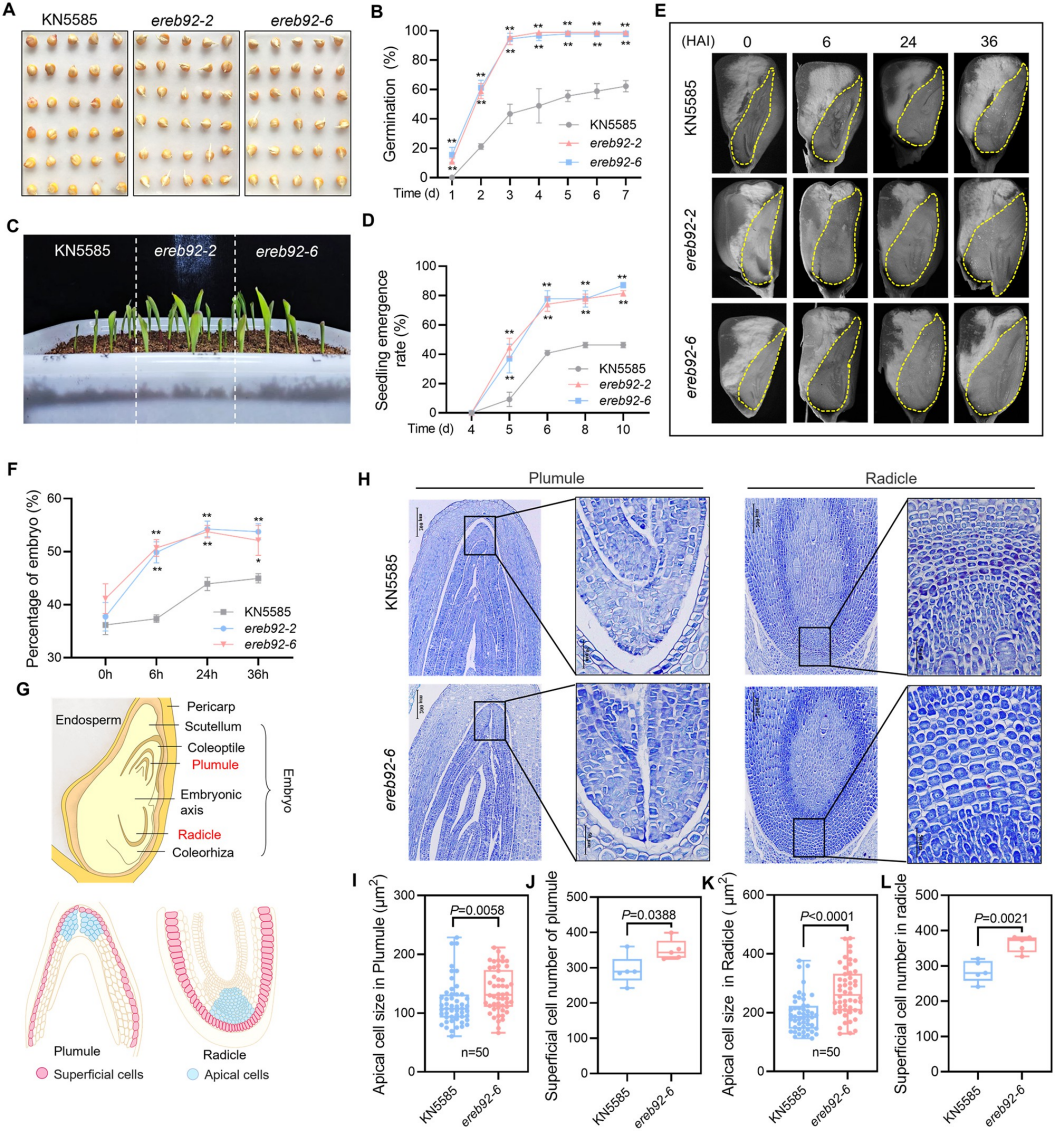
Loss-of-function of *ZmEREB92* enhanced seed germination in maize by promoting embryo growth during imbibition. (**A**) The germination performance for KN5585 and *ereb92* mutants (*ereb92-2* and *ereb92-6*) at 5 days after imbibition (DAI) on the filter paper infiltrated with sterile water. (**B**) The time-course germination of KN5585 and *ereb92* mutants for 1–7 DAI under normal condition. Error bars indicate mean ± SE (n = 3). (**C**) The phenotype of seedling emergence of KN5585 and ereb92 mutants after 10 days sowing in the soil. (**D**) The seedling emergence rates of KN5585 and *ereb92* mutants were counted at 4, 5, 6, 8, 10 days after sowing in the soil. Error bars indicate mean ± SE (n = 3). (**E**) The longitudinal section of KN5585 and *ereb92* mutant seeds at 0, 6, 24 and 36 HAI. The embryo region was marked with yellow dash line. (**F**) The percentage of embryo for KN5585 and *ereb92* mutant seeds at 0, 6, 24 and 36 HAI. The relative embryo proportion is calculated with the formula: Embryo area/ whole seed area *100%. The areas were calculated by ImageJ software. Error bars indicate mean ± SE (n = 5). (**B**, **D**, **F**) Asterisks indicate significant difference from the control (KN5585) at each time point (Two-way ANOVA followed by Tukey test, **P <0.01). (**G**) The schematic longitudinal view of the embryo structure in maize. (**H**) The histological sectioning and cytological analysis of the radicle and plumule region of KN5585 and *ereb92-6* seeds at 36 HAI. (**I**, **K**) The cell size in radicle (**I**) and plumule (**K**) of KN5585 and *ereb92-6* indictaed in (**G**), respectively. A total of 50 cells from three different sections of each line were counted. (**J**, **L**) The cell number of radicle and plumule of KN5585 and *ereb92-6* seeds indicated in (**G**). (**G-J**) The distribution of cell size is displayed by boxplot. Error bars indicates the value range and the box shows the medium and the upper and lower quartiles. The circles represent for individual datapoints of biological replicates in each line. Number marked for each data is the exact *P* value (Student’s *t*-test, *P<0.05, **P<0.01).

ZmEREB92 has been reported to regulate maize terpenoid phytoalexins previously [30]. Here, we observed that the Crispr/Cas9-mediated knockout mutants of *ZmEREB92* we generated before exhibited stronger seed germination as well as seedling emergence than wild type KN5585 (Fig 1A–1G). Such superiority can be retained in seeds harvested from different cultivation areas (S1 Fig). Radicle emergence is known as the determinant event for seed germination, which is dependent on elongating of embryo and loosening of surrounding structures [31]. By observing the longitudinal section of imbibed seeds, we found that the percentage of embryo was markedly higher in *ereb92* mutants at 6, 24 and 36 hours after imbibition (HAI) compared to those of KN5585, while no difference was found in quiescent embryos at 0 HAI (Fig 1E and 1F). Besides, similar pericarp thickness and water absorption rates were detected in KN5585 and *ereb92* mutant seeds (S2 Fig), indicating that rather than hydrate-mediated embryo swelling, endogenous embryo growth potential might be the primary force to drive faster germination of *ereb92* mutants.

To dissect the mechanisms by which ZmEREB92 regulates embryo enlargement during imbibition, we investigated the number of superficial cells and size of the apical cells in both radicle and plumule of KN5585 and *ereb92-6* seeds at 36 HAI (Fig 1G and 1H). In radicle, the average size of apical cells of *ereb92* mutants was enlarged by 39.2% and the number of superficial cells also significantly increased by 27.2% compared with those of KN5585 (Fig 1I and 1J). Similar results were also observed in plumule cells with an increment of 18.1% in the size of the apical cells and 19.5% in number of superficial cells by mutation of *ZmEREB92* (Fig 1K and 1L). These results demonstrated that the enhanced embryo growth was contributed by both cell expansion and cell division.

### Transcriptomics reveal the role of ZmEREB92 in regulating hormone-related pathways in imbibed seeds

**Fig 2:**
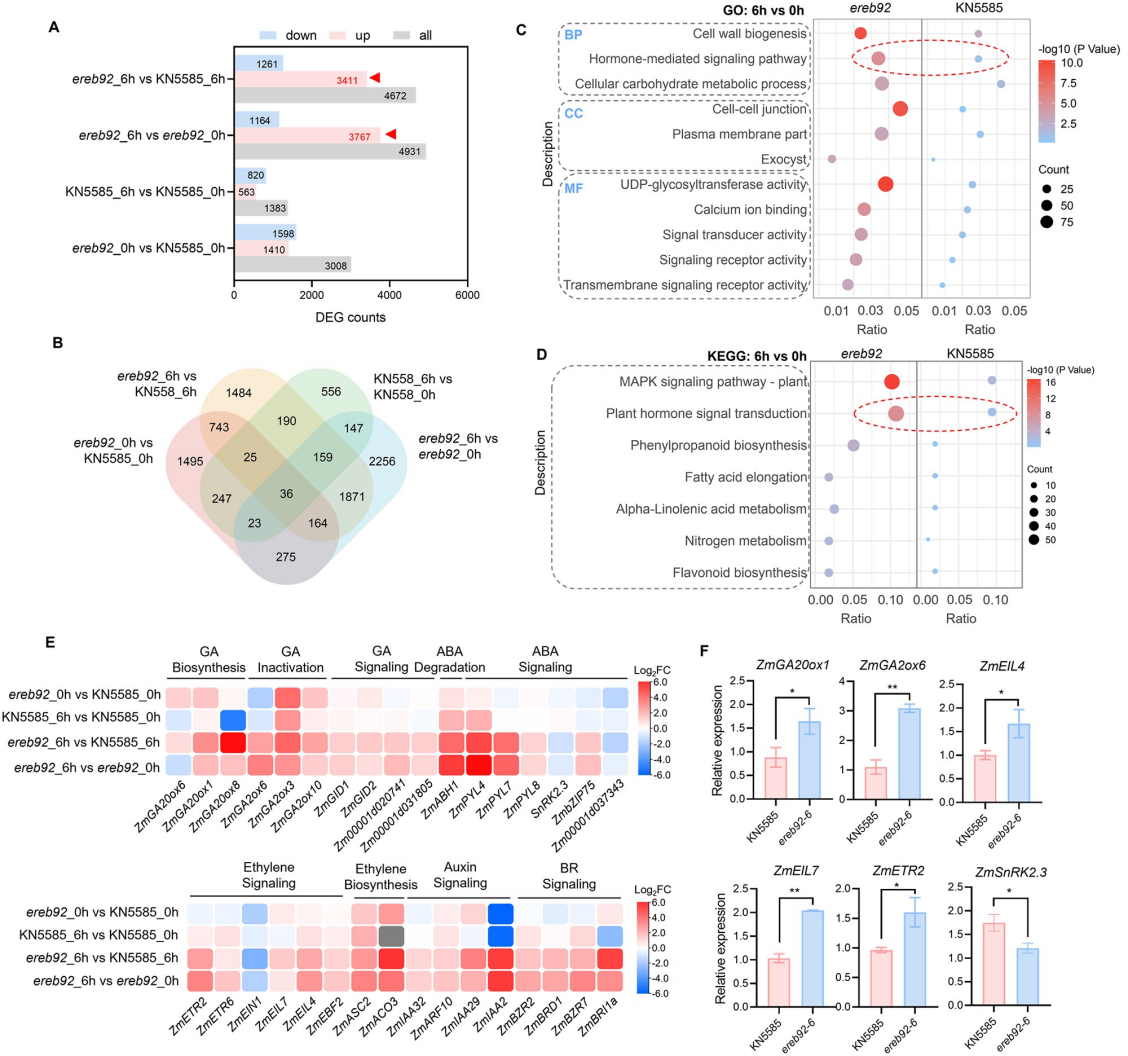
Transcriptomic profile reveals the role of ZmEREB92 in regulating hormone-related pathways. (**A**, **B**) A and B, Number of DEGs (Log_2_ (|FC|) ≥1, *P*<0.05) (**A**) and the Venn diagram shows the number of overlapped DEGs (**B**) in the comparison groups of *ereb92*_6h vs KN5585_6h, *ereb92*_6h vs *ereb92*_0h, KN5585_6h vs KN5585_0h and *ereb92*_6h vs KN5585_6h. Three independent experiments were performed for each sample at each time point. (**C**, **D**) GO (**C**) and KEGG (**D**) analysis of the DEGs in the comparison groups of *ereb92*_6h vs *ereb92*_0h and KN5585_6h vs KN5585_0h. (**E**) The heatmap shows the log_2_FC (Fold change) of differentially expressed hormone-related genes in the comparison groups of *ereb92*_6h vs *ereb92*_0h or *ereb92*_6h vs KN5585_6h. (**F**) qPCR analysis for several selected hormone-related genes in seeds of *ere92* mutant and KN5585 at 6 HAI. Error bars indicate mean ± SE (n = 4). *Ef1a* was used as the reference gene and relative expression level was normalized to one biological replicate of KN5585. Asterisks indicate significant difference (Student’s *t*-test, *P<0.05, **P<0.01).

To understand the regulatory network of ZmEREB92, we employed RNA-seq using the seeds at 0 and 6 HAI of KN5585 and *ereb92-6* mutant (S1 Data). Compared to 0 HAI, more than a thousand of differentially expressed genes (DEGs) were detected at 6 HAI in both KN5585 and *ereb92* mutant seeds (Fig 2A). Notably, the DEGs were mostly enriched in the up-regulated genes in comparison group of *ereb92*_6h vs KN5585_0h (3411 genes) and *ereb92*_6h vs *ereb92*_0h (3767 genes) (Fig 2A and 2B), suggesting that the release of suppression on downstream genes by *ZmEREB92* mutation might play a major role in facilitating seed germination. Further GO and KEGG analysis of these up-regulated DEGs were all revealed a significant enrichment in hormone-related pathways (Fig 2C and 2D, S3A and S3B Fig, S1 and S2 Tables). Besides, other pathways including cell wall biogenesis, carbohydrate metabolism, UDP-glycosyltransferase activity and MAPK signaling were also enriched (Fig 2C and 2D, S3A and S3B Fig, S1 and S2 Tables). Together, these results strongly imply the role of hormone signaling in ZmEREB92-mediated seed germination.

We thus analyzed the hormone-related DEGs in *ereb92*_6h vs *ereb92*_0h or *ereb92*_6h vs KN5585_6h (Fig 2E and S3 Table). Among them, GA biosynthesis and inactivation genes were both up-regulated in *ereb92*_6h compared to *ereb92*_0h or KN5585_6h (Fig 2E). Such increased expression pattern was also detected for several GA signaling genes, including GA receptor genes *ZmGID1* and *ZmGID2* (Fig 2E), suggesting a more active GA metabolism and signaling in imbibed *ereb92* mutant seeds. Besides, the expression of *ZmABH1*, the ABA catabolic gene, was largely increased in *ereb92*_6h compared to *ereb92*_0h or KN5585_6h (Fig 2E). Three *PYL* genes (ABA receptor genes) and a set of *PP2C* genes, the negative regulator of ABA signaling, were also showed increased abundance, while ABA signaling gene *ZmSnRK2*.*3* was slightly down-regulated (Fig 2E and S3C Fig). Besides, multiple ethylene, auxin and brassinosteroid (BR) related genes were also up-regulated except for *ZmEIN1* (Fig 2E and S3C Fig), suggesting these pathways might contribute to the enhanced germination in *ereb92* mutant, too. Further q-PCR analysis confirmed our RNA-seq results, in which *ZmGA20ox1*, *ZmGA2ox6*, *ZmEIL4*, *ZmEIL7* and *ZmETR2* were up-regulated in *ereb92* mutant at 6 HAI compared to those of KN5585, while *ZmSnRK2*.*3* expression was reduced (Fig 2F).

The AP2/ERF family members are known to bind the GCC-box in target gene promoters [32]. We next performed *cis*-element enrichment analysis to investigate whether ZmEREB92-triggered transcriptomic changes are mainly dependent on the GCC-box. We divided the datasets from KN5585_6h vs 0h (S1) and *ereb92*_6h vs 0h (S2) into three groups, including all differentially expressed genes (ADEG), up-regulated genes (URG) and down-regulated genes (DRG) (S4A Fig). Non-differentially expressed genes (NDEG) were also analyzed as the negative control. We firstly scanned the promoters (1.5 kb upstream of ATG) of ADEG, URG and DRG in S2 and calculated the percentage of genes in each group that contains a certain Transcription factor binding site (TFBS) in their promoters. The TOP10 enriched TF families were shown and we found that in the ADEG group, only TALE was enriched higher than NDEG (S4B Fig). For the URG group, the ERF, TALE and LBD displayed higher enrichment than NDEG, among which ERF accounts for the highest proportion (S4C Fig). However, in the DRG group, no TF family showed higher percentage than NDEG (S4D Fig). We further analyzed the genes that are presented in S2 but not in S1 (S2-S1) and got similar results (S4E–S4G Fig). Together, we suggested that the GCC-box might have the major effect in transcriptional up-regulation rather than down-regulation in the *ereb92* mutant during imbibition.

### Ethylene signaling plays a major role to drive seed germination in *ereb92* mutants

**Fig 3:**
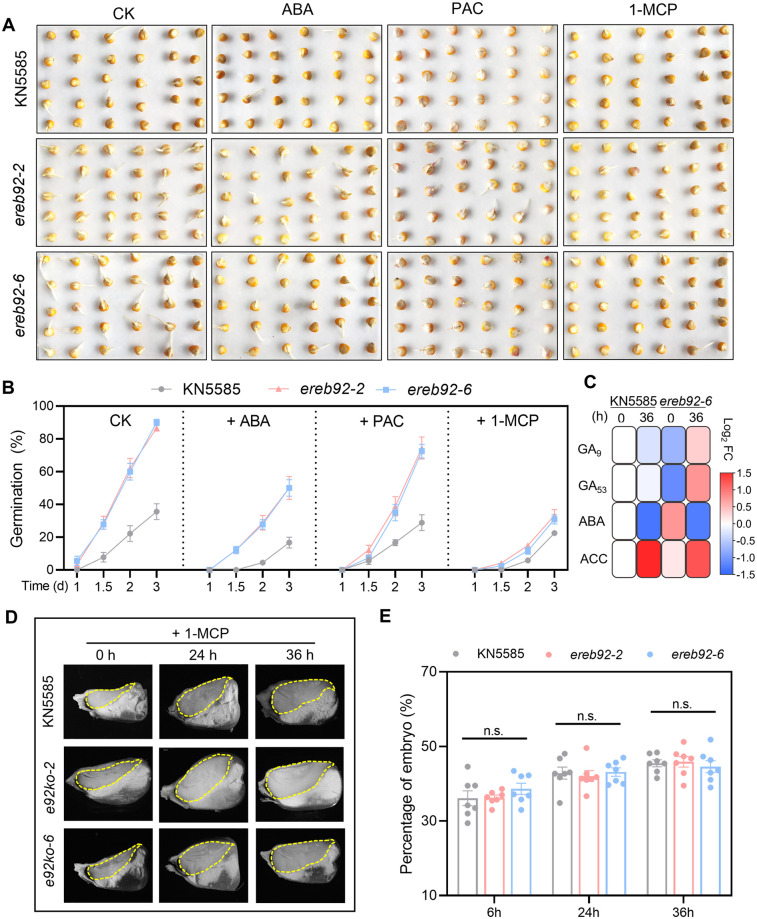
Ethylene signaling plays a major to promote seed germination in *ereb92* mutants. (**A**) The germination performance at the for KN5585 and *ereb92* mutants at 3 DAI under normal condition (CK), 50 μM ABA treatment, 100 mg/L Paclobutrazol (PAC, inhibitor of GA biosynthesis) treatment and 200 mg/L 1-Methylcyclopropene (1-MCP, inhibitor of ET receptor). (**B**) The germination rate of KN5585 and *ereb92* mutants counted at 1, 1.5, 2 and 3 DAI under the treatments described in (A). Error bars indicate mean ± SE (n = 4). (**C**) The heatmap showed the Log_2_FC of the hormone level relative to KN5585_0h. (**D**) The longitudinal section of the seeds of KN5585 and *ereb92* mutants at 0, 24 and 36 HAI under 1-MCP treatment. Embryo region was sketched with yellow dash line. (**E**) The percentage of embryo for seeds of KN5585 and *ereb92* mutant at 0, 24 and 36 HAI under 1-MCP treatment. The relative embryo proportion is calculated by ImageJ software. The circles are represented for individual datapoints of biological replicates in each line. Error bars indicate mean ± SE (n = 7). n. s. indicates no significant difference using one-way ANOVA followed by Tukey tests (*P*>0.05).

Our RNA-seq data revealed that differentially expressed hormone-related genes were mostly enriched in GA, ABA and ethylene pathways (Fig 2), we next asked which hormone plays the main role. By applying hormone or corresponding inhibitor treatments, we firstly found that ABA application inhibited the germination of both KN5585 and *ereb92* mutants, but the disparity between them was still large (Fig 3A and 3B). As with ABA treatment, the paclobutrazol (PAC) treatment, which inhibits GA biosynthesis, also resulted in similar trends (Fig 3A and 3B). Notably, application of 1-Methylcyclopropene (1-MCP), the competitive inhibitor of ethylene receptor, almost eliminated the difference between KN5585 and *ereb92* mutants (Fig 3A and 3B), indicating a more important role for ethylene-related pathway. These results were also supported by *p*-value analysis (S5 Fig). However, the content of ethylene precursor 1-aminocyclopropanecarboxylic acid (ACC) in *ereb92-6* seemed slightly lower than that in KN5585 at 36 HAI. These results implicated that it might be ethylene signaling rather than ethylene biosynthesis that acts in ZmEREB92-regulated seed germination. Meanwhile, GA accumulation and ABA degradation were more intensive in *ereb92-6* mutant than KN5585 during imbibition, resulting in a significant reduction of ABA/GA ratio (Fig 3C and S6 Fig).

We then performed ethephon (ETH) and 1-MCP treatment on seeds of maize inbred line Mo17 to clarify the action of ethylene signaling in maize seed germination. Results showed that although ETH treatment only slightly elevated the germination rate at 2 HAI, 1-MCP treatment continuously inhibited seed germination (S7A and S7B Fig). The embryo proportion was also increased by ETH but decreased by 1-MCP treatment at 36 HAI (S7C and S7D Fig). These findings confirmed that ethylene signaling played a positive role in regulating maize seed germination. Similarly, 1-MCP treatment compromised the embryo enlargement in *ereb92* mutants after imbibition (Fig 3D and 3E). We also investigated whether the expression of *ZmEREB92* could be affected by exogenous ethephon (ETH) or 1-MCP treatment during imbibition. The results showed that *ZmEREB92* was significantly down-regulated by ETH at 24HAI but strongly induced by the 1-MCP at 12 and 24 HAI (S7E and S7F Fig). Altogether, we demonstrated that ZmEREB92 regulated seed germination by affecting the embryo growth, which might be dominantly mediated by ethylene signaling.

### ZmEREB92 directly represses the expression of *ZmEIL7*

**Fig 4:**
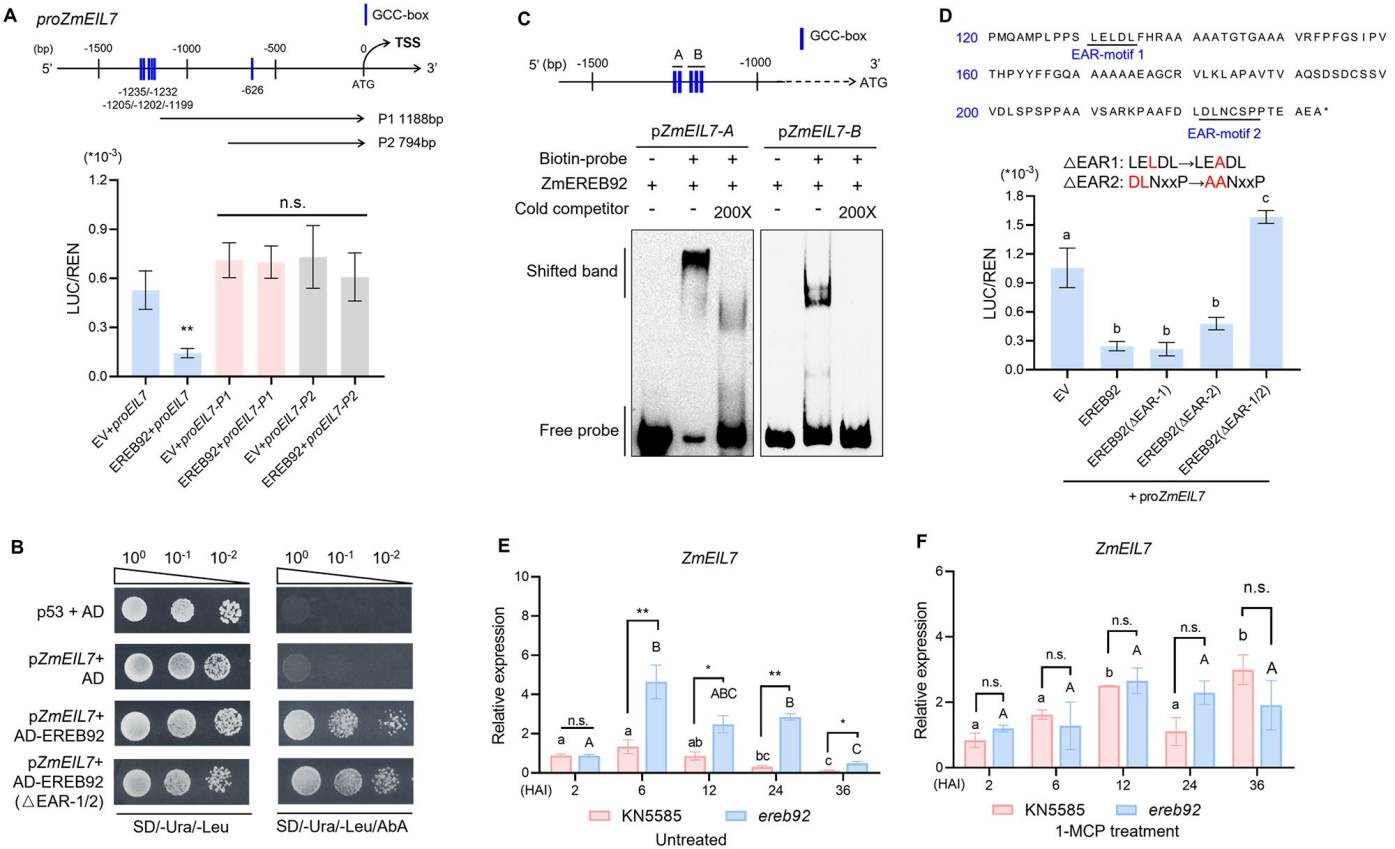
ZmEREB92 inhibits the transcription of *ZmEIL7* by directly binds to the G-boxes in *ZmEIL7* promoter. (**A**) Dual-Luciferase Reporter (DLR) assays in maize protoplast showing that ZmEREB92 negatively regulated *ZmEIL7* transcription through the -1188 to -1500 bp region upstream to the start codon. The different promoter region of *ZmEIL7* were co-transformed with empty vector (EV) or *ZmEREB92*. p35S-REN was used as the internal control. Error bars indicate mean ± SE (n = 3). Asterisks indicate significant difference (Student’s *t*-test, **P<0.01). (**B**) The repression of ZmEREB92 on *ZmEIL7* transcription was dependent on two EAR-motif at C terminus. The EAR-motif was indicated by black line. Single or double mutations of two EAR-motifs (ΔEAR-1, ΔEAR-2 and ΔEAR-1/2) in ZmEREB92 were generated and co-transformed with *ZmEIL7* promoter, respectively. Error bars indicate mean ± SE (n = 3). Different lowercase letters represent significant differences (one-way ANOVA followed by Tukey tests, *P*<0.05). (**C**) Yeast one hybrid assays suggest the direct binding of ZmEREB92 to ZmEIL7 promoter. The promoter of *ZmEIL7* was co-transformed with AD-ERBE92 or AD-EREB92(ΔEAR1/2) and grown on selective medium (SD/-Ura/-Leu/AbA). AbA, Aureobasidin, 400 ng·mL^-1^. Empty pGADT7 vector (AD) was also co-transformed as the negative control. (**D**) EMSA to show the direct binding of ZmEREB92 to the GCC-boxes in *ZmEIL7* promoter. Two fragments containing GCC-boxes in *ZmEIL7* promoter were labeled with biotin and incubated with ZmERBE92 purified recombinant proteins. 200-fold excess of unlabeled probes were used for competition. (**E**, **F**) The expression pattern of ZmEIL7 in KN5585 and *ereb92-6* mutant at 2, 6, 12, 24 and 36 HAI under control (**E**) and 1-MCP treatment (**F**). Error bars indicate mean ± SE (n = 3). *Ef1a* was used as the reference gene and relative expression level was normalized to one biological replicate of 2 HAI of KN5585. Different lowercases or majuscules represent significant difference in KN5585 or *ereb92-6* mutant, respectively (one-way ANOVA followed by Tukey tests, *P*<0.05). Asterisks indicate significant difference between KN5585 and *ereb92-6* mutant at each time point (Student’s *t*-test, **P<0.01, n.s. no significant difference).

Among those up-regulated ethylene-related genes in our RNA-seq data, the promoter of *ZmEIL7* contains most abundant GCC-boxes (S8A Fig). To verify whether *ZmEIL7* is a target gene of ZmEREB92, the 1500 bp fragment upstream the start codon was amplified and subjected to a dual-luciferase (LUC) reporter (DLR) assay (S8B Fig). In line with the increased expression of *ZmEIL7* in *ereb92* mutant, *ZmEIL7* promoter activity was significantly decreased in the presence of ZmEREB92 (Fig 4A). Such suppression was released by deleting the GCC-box enriched region in the promoter (*proEIL7-P1*) or retain only 794 bp upstream fragment (*proEIL7-P2*) (Fig 4A), suggesting that the GCC-box enriched region might be the site regulated by ZmEREB92. Yeast-one-hybrid (Y1H) assays were conducted subsequently to reveal the direct binding of ZmEREB92 to *ZmEIL7* promoter containing the GCC-box enriched region (Fig 4B). Further EMSA confirmed that the GCC-box enriched region (-1235 to -1194 bp) served as the binding site for ZmEREB92 (Fig 4C). ZmEREB92 was found to lack trans-activation activity in our previous work [30]. Sequence analysis showed that ZmEREB92 contains two EAR motifs at C terminus (Fig 4D), which confer the repression activity by interacting with co-repressors, such as TOPLESS protein [33]. Mutation of either EAR motif-1 or -2 was insufficient to affect *proZmEIL7* activity, while double mutations of both EAR-motifs resulted in a complete loss of suppression on *ZmEIL7* promoter (Fig 4D), indicating that the transcriptional repression of ZmEREB92 on *ZmEIL7* depends on both EAR motifs. Besides, the mutation of EAR-motifs did not impair the binding of ZmEREB92 to *ZmEIL7* promoter (Fig 4B).

Expression analysis revealed that *ZmEIL7* expressed steadily within 12 HAI and decreased from 24 to 36 HAI in KN5585. In contrast, it was increased from 6 to 24 HAI in *ereb92-6*, both of which were significantly higher than those in KN5585 (Fig 4E), but decreased at 36 HAI. However, under 1-MCP treatment, not only the induction of *ZmEIL7* in *ereb92* mutant was abolished, but also the difference between KN5585 and *ereb92* mutant was eliminated (Fig 4F). Phylogenetic tree of all EIL members in maize, rice and Arabidopsis showed that ZmEIL7 has a closest relationship with rice OsEIL6 (S9A Fig), and several GCC-boxes were also found in the promoter of *OsEIL6* (S10B Fig), but its function remains unclear. Together, we illustrated that ZmEREB92 directly binds to the GCC-box enriched region of *ZmEIL7* promoter to suppress its expression, such inhibition is dependent on two EAR motifs at C-terminus of ZmEREB92.

### α-amylase contributes to ethylene-mediated germination in *ereb92* mutants by accelerating starch mobilization

**Fig 5:**
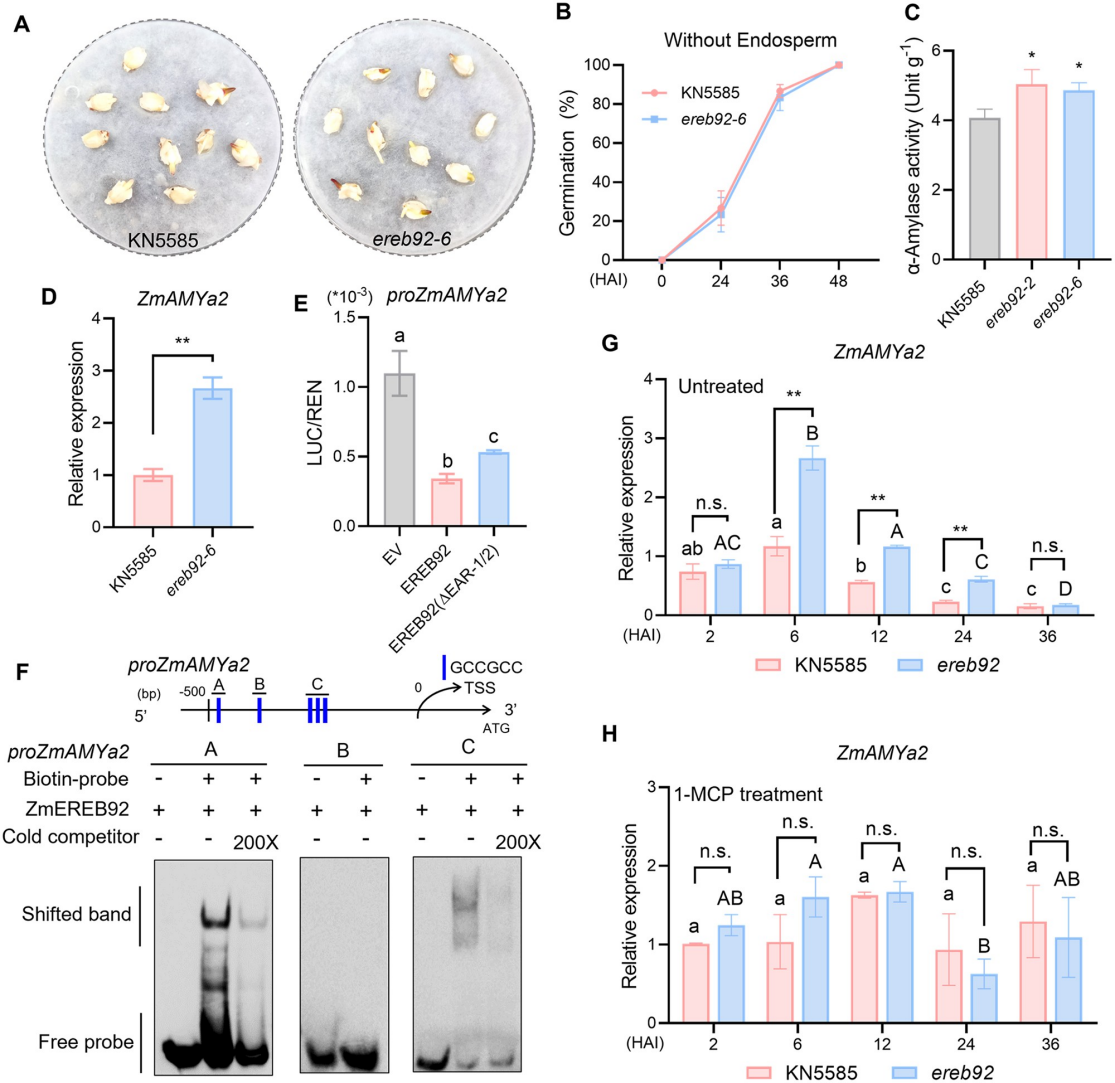
ZmEREB92 directly inhibit *ZmAMYa2* transcription and resulted in accelerated starch mobilization in imbibed seeds of *ereb92* mutants. (**A**) The germination performance at 24 HAI using separated embryos of KN5585 and *ereb92-6* mutant. (**B**) The germination rate at 0, 24, 36 and 48 HAI of KN5585 and *ereb92-6* mutant using separated embryos. Error bars indicate mean ± SE (n = 3). (**C**) α-amylase activity in KN5585 and *ereb92* mutants at 24 HAI. Error bars indicate mean ± SE (n = 7). Asterisks indicate significant difference (Student’s *t*-test, *P<0.05). (**D**) The relative expression of *ZmAMYa2* in *ereb92-6* mutant and KN5585 at 6 HAI. Error bars indicate mean ± SE (n = 3). *Ef1a* was used as the reference gene and relative expression level was normalized to one biological replicate of KN5585. Asterisks indicate significant difference (Student’s *t*-test, **P<0.01). (**E**) DLR assays in maize protoplast showing that ZmEREB92 negatively regulated *ZmAMYa2* transcription and such repression was partially dependent on two EAR-motifs. The promoter of *ZmAMYa2* were co-transformed with EV, ZmEREB92 or ZmEREB92(ΔEAR1/2), respectively. p35S-REN was used as the internal control. Error bars indicate mean ± SE (n = 3). Different lowercases indicate significant difference (one-way ANOVA followed by Tukey tests, *P*<0.05). (**F**) EMSA to show the direct binding of ZmEREB92 to the GCC-boxes in A and C fragments of *ZmAMYa2* promoter. Three fragments containing GCC-boxes in *ZmAMYa2* promoter were labeled with biotin and incubated with ZmERBE92. 200-fold excess of unlabeled probes were used for competition. (**G**, **H**) The expression pattern of *ZmAMYa2* in KN5585 and *ereb92-6* at 2, 6, 12, 24 and 36 HAI under control (**G**) and 1-MCP treatment (**H**). Error bars indicate mean ± SE (n = 3). *Ef1a* was used as the reference gene and relative expression level was normalized to one biological replicate of 2 HAI of KN5585. Different lowercases or majuscules represent significant difference in KN5585 or *ereb92-6* mutant, respectively (one-way ANOVA followed by Tukey tests, *P*<0.05). Asterisks indicate significant difference between KN5585 and *ereb92-6* mutant at each time point (Student’s *t*-test, **P<0.01, n.s. no significant difference).

With findings that the *ereb92* mutants possess more active embryo after imbibition (Fig 1C and 1D), we are curious whether this growth potential is derived from embryo itself. To answer this question, we performed germination assay using separated embryos without endosperm. Both KN5585 and *ereb92-6* mutant seeds germinated rapidly with an identical germination tendency (Fig 5A and 5B), suggesting that the growth potential might also contributed by endosperm. Starch degradation in starchy endosperm is known to provide carbohydrate and energy for seed germination and early growth [8]. Indeed, the α-amylase activities of *ereb92* mutants were significantly higher than that of KN5585 (Fig 5C). Two amylase genes, *ZmAMYa2* and *ZmAMYb5* were identified as DEGs from our RNA-seq data (S3C Fig). Among them, the promoter of *ZmAMYa2* contains an array of five GCC-boxes while *ZmAMYb5* contains none (S10A Fig). Further q-PCR assays confirmed that the expression of *ZmAMYa2* was higher in *ereb92* mutant seeds at 6 HAI (Fig 5D).

Subsequent DLR assays demonstrated that *proZmAMYa2* activity was inhibited by the presentation of ZmEREB92 (Fig 5E). However, the double mutations of two EAR-motifs in ZmEREB92 only partially rescued the activity of *proZmAMYa2* (Fig 5E), suggesting involvement of other repressors. The EMSA results further established that ZmEREB92 directly binds to the A and C regions of *proZmAMYa2* (Fig 5F). By q-PCR analysis, we found that *ZmAMYa2* shared a similar expression pattern with *ZmEIL7*. Phylogenetic analysis showed ZmAMYa2 is closely related to RAmy3D in rice (S9B Fig), which has a positive role in seed germination by facilitating starch degradation in rice [14]. A series of GCC-boxes were also enriched in the promoter of *RAmy3D* (S10B Fig), suggesting similar regulation of this gene by ERF transcription factors. Together, ZmEREB92 also regulates the starch mobilization during imbibition to affect seed germination, which is possibly mediated by the direct repression of ZmEREB92 on *ZmAMYa2* expression.

### Rice ortholog of ZmEREB92 is functionally conserved in regulating seed germination

**Fig 6:**
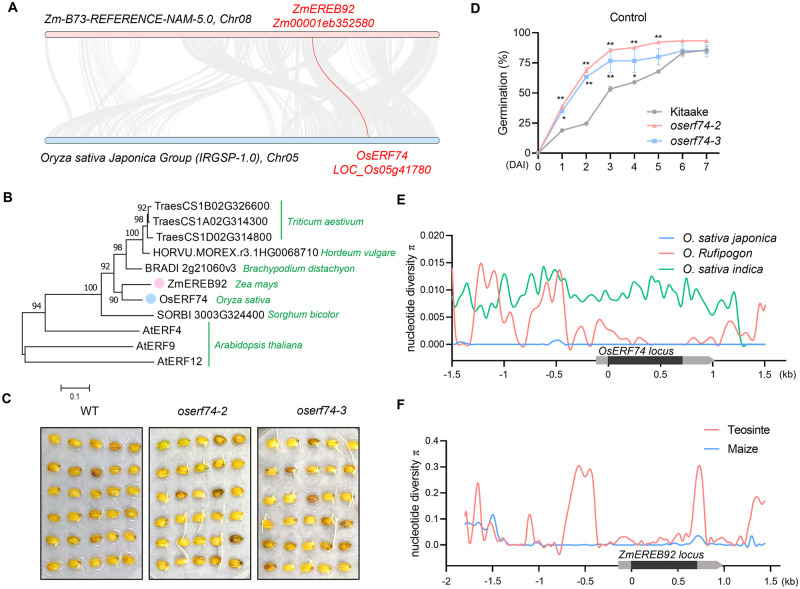
Rice ortholog of *ZmEREB92* negatively regulates seed germination and underwent selection during rice domestication. (**A**) Syntenic analysis of *ZmEREB92* and *OsERF74* from maize chromosome 8 (*B73 RefGen_v5*, pink) and rice chromosome 5 (Nipponbare IRGSP-1.0, light blue), respectively. (**B**) Phylogenetic analysis of ZmEREB92 and its orthologs in major cereal crops and Arabidopsis using neighbor-joining method. The numbers shown next to the branches indicate bootstrap values from 1000 replicates. (**C**) The germination performance of Kitaake and *Oserf74* mutants at 3 DAI under normal condition (25 °C). (**D**) Time-course seed germination rate from 0–7 DAI for Kitaake and *Oserf74* mutants under normal condition. Error bars indicate mean ± SE (n = 3). Asterisks indicate significant difference from the control (Kitaake) at each time point (Two-way ANOVA followed by Tukey test, *P <0.05, **P <0.01). (**E**) Nucleotide diversity across the *OsERF74* locus among *O*. *sativa japonica* (blue), *O*. *sativa indica* (green) and *O*. *Rufipogon* (pink). The genomic region of *OsERF74* is shown at the X axis. (**F**) Nucleotide diversity across the *ZmEREB92* locus among maize (blue) and teosinte (pink). The genomic region of *ZmEREB92* is shown at the X axis.

Among all ERF VIII subgroup members in maize, rice and Arabidopsis, ZmEREB92 is phylogenetically closest to rice OsERF74 (S11 Fig). Syntenic analysis confirmed that *OsERF74* is the ortholog of *ZmEREB92* in rice (Fig 6A). Phylogenetic tree of *ZmEREB92* and its other orthologs from major cereals and Arabidopsis also clustered ZmEREB92 and OsERF74 into one branch (Fig 6B). *ZmEREB92* showed a maximum expression in embryo during kernel maturation, while *OsERF74* expressed highest in reproduction organs, followed by endosperm during seed development (S12 Fig). To test whether OsERF74 exerts similar functions as ZmEREB92, we generated the Crispr/Cas9-mediated knockout mutants of *OsERE74* (S13 Fig). Two independent mutant lines were obtained and showed enhanced seed germination performance (Fig 6C and 6D). A genome-wide scan for genes that underwent convergent selection in rice and maize domestication identified *OsERF74* as a selected gene on chromosome 5 [34]. Consistent with this, the nucleotide diversity of *OsERF74* promoter region was strikingly reduced in *Oryza sativa ssp*. *japonica* (hereafter, *japonica*) compared to wild rice *Oryza rufipogon* (hereafter, *rufipogon*), while genomic region of *OsERF74* showed an increased diversity in *Oryza sativa ssp*. *indica* (hereafter, *indica*) (Fig 6E). *ZmEREB92* was skipped in previous studies because it was not assembled in *B73 RefGen_v4* (S14 Fig and S4 Table). We analyzed the nucleotide diversity across *ZmEREB92* locus using a small maize and teosinte panel based on the public genome assembled by *B73 RefGen_v5*. As with *OsERF74*, similar loss of nucleotide diversity was detected in the promoter region of *ZmEREB92* (Fig 6F).

We next profiled the expression levels of *ZmEREB92* and *OsERF74* in different maize and rice subspecies using the public high-throughput transcriptome datasets. The results revealed that *ZmEREB92* is significantly down-regulated in maize compare to those in teosinte (S15A Fig) [35]. Additional analysis in 368 maize inbred lines showed that the temperate maize lines tend to have lower expression level of *ZmEREB92* than tropical maize lines (S15B Fig) [36]. Besides, no difference was found between the stiff stalk (SS) and non-stiff stalk (NSS) heterotic groups of temperate maize (S15B Fig), suggesting that we should utilize other germplasm resources such as tropical/subtropical lines to explore the potential excellent alleles of *ZmEREB92* for breeding programs. In rice, *OsERF74* showed a lower expression in *temperate japonica* than those in *rufipogon*, *indica* and *tropical japonica* (S15C Fig) [37]. Using other transcriptomic datasets for *indica* and *japonica*, we found that *OsERF74* displayed higher expression in *indica* compare with *japonica* (S15D Fig) [38,39], among which *indica* are usually distributed in tropical regions while *japonica* are mainly grown in temperate regions [40]. Such pattern is similar with the expression divergence of *ZmEREB92* in temperate and tropical maize (S15B Fig). Our results suggest that the orthologous gene pair *ZmEREB92/OsERF74* might have experienced convergently selection during maize and rice domestication with conserved function in seed germination control and show similar expression divergence.

### ZmEREB92 and OsERF74 conservatively regulate the sensitivity to temperature during seed germination

**Fig 7:**
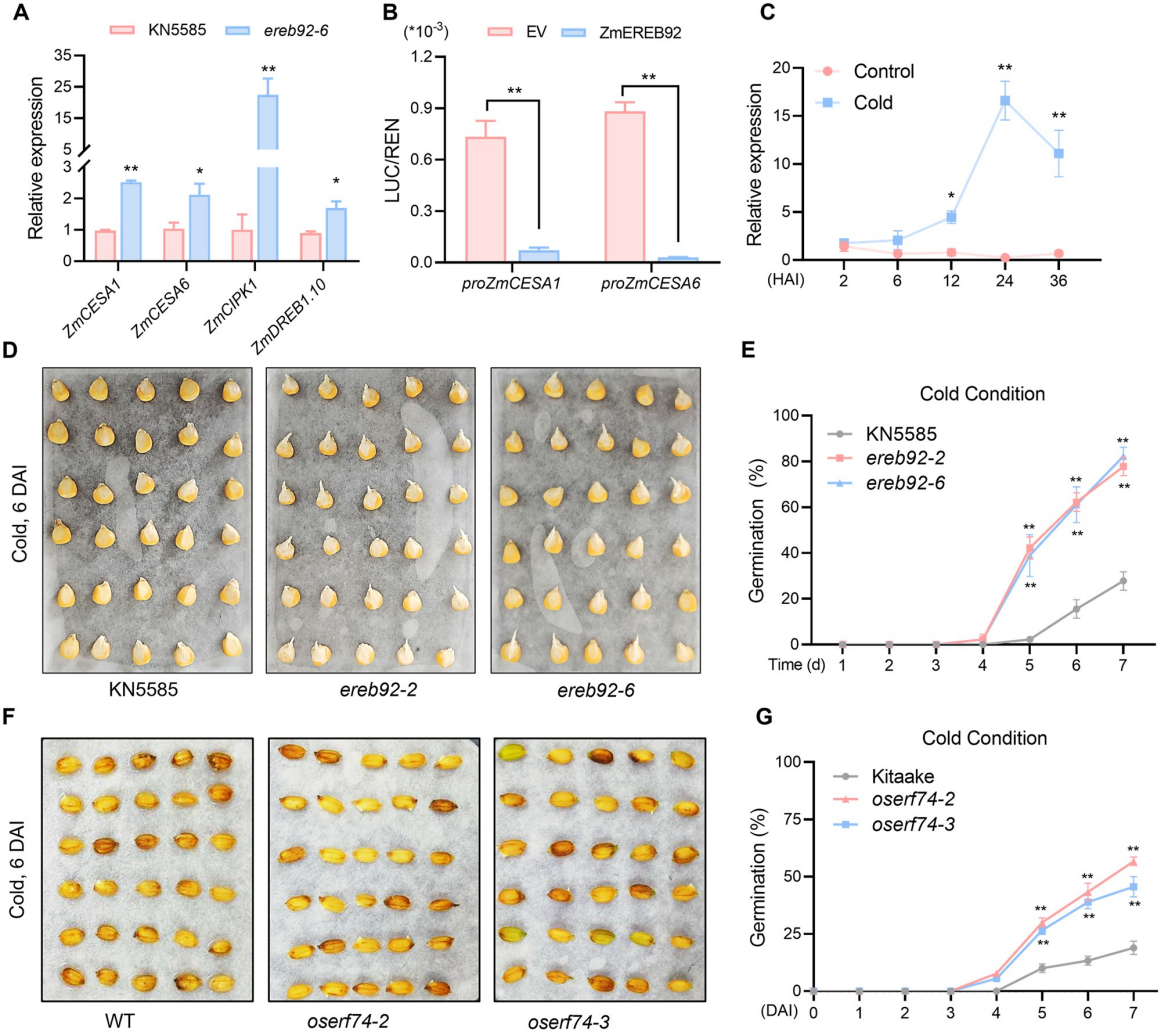
The orthologous gene pair *ZmEREB92/OsERF74* show conserved functions in seed germination under cold stress. (**A**) Relative expression analysis of cold-related genes in seeds of *ere92-6* mutant and KN5585 at 6 HAI. *Ef1a* was used as the reference gene and relative expression level was normalized to one biological replicate of KN5585. (**B**) DLR assays for the promoters of *ZmCESA1* and *ZmCESA6* co-transformed with EV or ZmEREB92. (**C**) The expression pattern of *ZmEREB92* during imbibition under normal (28 °C) and cold (12 °C) condition, respectively. *Ef1a* was used as the reference gene and relative expression level was normalized to one biological replicate of control-2HAI. (**A**-**C**) Error bars indicate mean ± SE (n = 3). Asterisks indicate significant difference (Student’s *t*-test, *P<0.05, **P<0.01). (**D**) The seed germination performance of KN5585 and *ereb92* mutants at 6 DAI under cold condition (12 °C). (**E**) Time-course seed germination rate from 1–7 DAI for KN5585 and *ereb92* mutants. Error bars indicate mean ± SE (n = 3). (**F**) The germination performance of Kitaake and *Oserf74* mutants at 6 DAI under cold condition (15 °C). (**G**) Time-course seed germination rate from 0–7 DAI for Kitaake and *Oserf74* mutants under cold condition. Error bars indicate mean ± SE (n = 3). (**E**, **G**) Asterisks indicate significant difference from the control (Kitaake) at each time point (Two-way ANOVA followed by Tukey test, **P <0.01).

Temperature is an essential environmental signal that sensed by seed to determine germination [41]. Previous studies revealed that both *ZmEREB92* and *OsERF74* could be induced by cold [42,43]. In our transcriptome data, a series of cold-related genes were up-regulated in *ereb92* mutant, including *CESAs*, *DREBs* and *CIPKs* (S16 Fig). The q-PCR results confirmed that these genes expressed higher in *ereb92* mutant seeds at 6 HAI (Fig 7A). Several GCC-boxes were found in the promoter region of these two genes and DLR assays proved that the transcription of both *CESA* genes can be strongly repressed by ZmEREB92 (Fig 7B and S10C Fig). These results imply that ZmEREB92 might also regulate seed germination under cold condition. To verify this, we firstly demonstrated that the expression of *ZmEREB92* was indeed up-regulated by cold treatment but repressed in normal condition during imbibition (Fig 7C). We then observed a much stronger seed germination and seedling establishment for *ereb92* mutants compared to KN5585 in cold condition (Fig 7D and 7E and S17 Fig), while such superiority was not detected under PEG treatment (S18A and S18B Fig). The expression of *ZmEREB92* was only slightly induced at 12HAI but reduced to the control level at 24 and 36 HAI by PEG treatment (S18C Fig). Our results suggest a specific role for ZmEREB92 in cold tolerance. In rice, the mutation of *OsERF74* also resulted in enhanced seed germination under cold stress (Fig 7F and 7G), suggesting conserved function for orthologous *ZmEREB92/OsERF74* to regulate seed germination in a temperature-sensitive manner.

## Discussion

**Fig 8:**
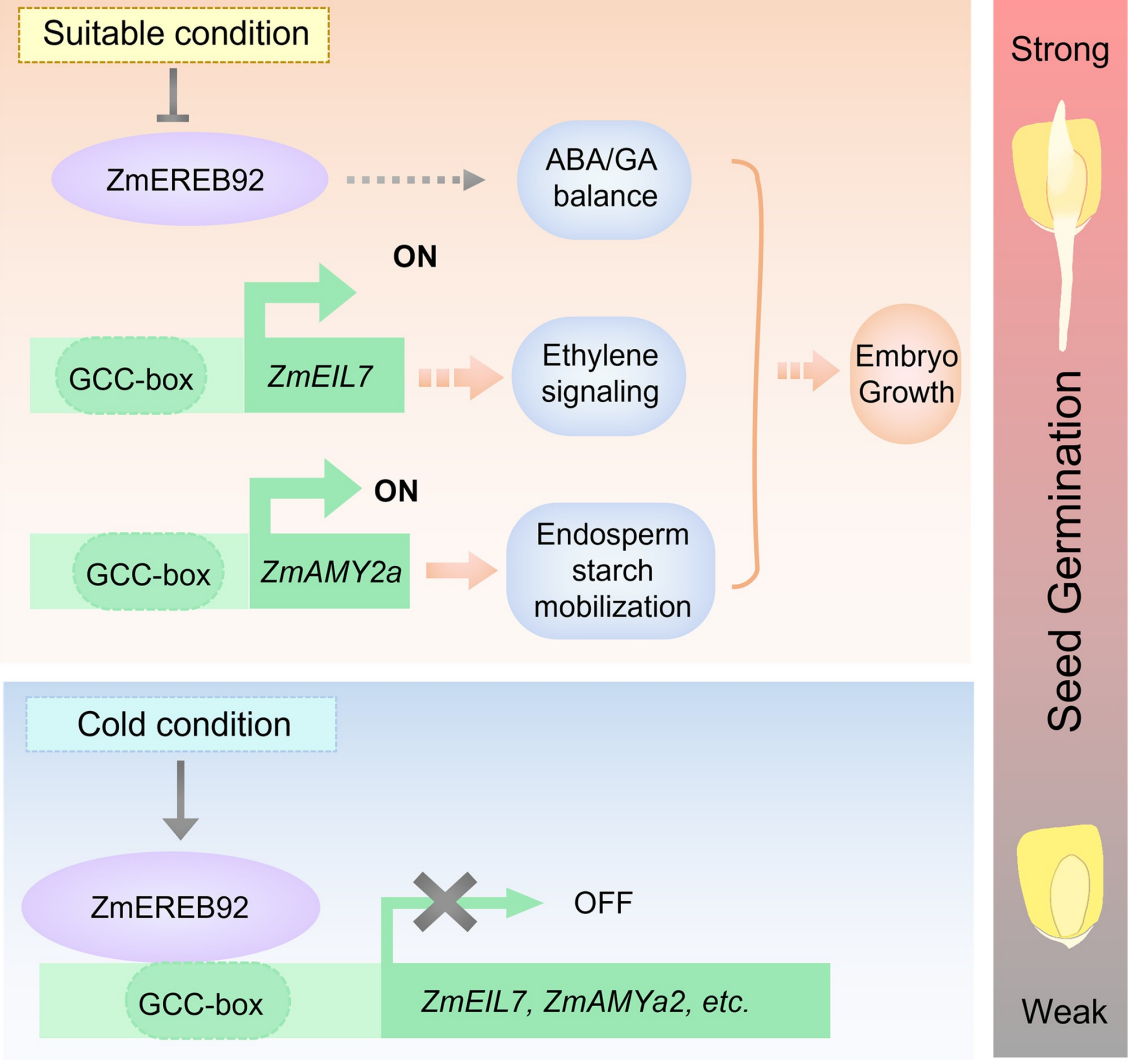
Proposed model for the temperature-sensitive regulation on seed germination by ZmEREB92. When maize seeds are germinating in normal condition, inhibited expression of *ZmEREB92* leads to the release of *ZmEIL7* and *ZmAMYa2* transcription, which enhance ethylene signaling and endosperm starch degradation, thereby promoting embryo growth and ensuring timely germination for maize seeds. When the condition is cold, the expression of *ZmEREB92* is drastically induced in imbibed seeds, which in turn suppresses the transcription of *ZmEIL7* and *ZmAMYa2* by directly binding to the GCC-box enriched region in their promoters, ultimately resulting in blocked seed germination.

Synchronous germination is crucial basis to ensure high crop yield. As the thermophilic plant, maize constantly confronts cold stress when sown in spring, which seriously hampers germination performance and seedling growth [5]. Thus, it has become an important goal to control seed germination in maize breeding, in which identification for key regulators is urgently needed. Our current study reveals the function of ZmEREB92 as a negative regulator of maize seed germination in a temperature-sensitive manner (Fig 8). In suitable condition, *ZmEREB92* is transcriptionally inhibited, resulting in enhanced expression of *ZmEIL7* and *ZmAMYa2*, which in turn promote the ethylene signaling and starch mobilization to contribute to timely germination (Fig 8). When the condition is cold, the expression of ZmEREB92 was strongly induced, which repress the expression of *ZmEIL7* and *ZmAMYa2* by directly binding to the GCC-box enriched region of their promoters, thus leading to inhibited seed germination (Fig 8). A similar function has been illustrated for *OsERF74*, the ortholog of *ZmEREB92* in rice. Reduced nucleotide diversity was detected in the promoter regions of both *ZmEREB92* and *OsERF74*, suggesting possible convergently selection of this orthologous gene pair during maize and rice domestication (Fig 6).

Plant hormones play as intrinsic drivers to control seed germination. Apart from the central role of ABA/GA balance in regulating germination, ethylene is also considered as an important facilitator of seed germination since 1960s [44,45]. Although our transcriptomic analysis showed that a plenty of DEGs were enriched in ABA, GA and ethylene-related pathways, the 1-MCP treatment, to a greater extent, narrowed the seed germination disparity between *ereb92* mutants and KN5585 compared to ABA or PAC treatment (Figs 2E, 3A and 3B and S5 Fig), indicating that ethylene signaling plays a major role to promote germination in *ereb92* mutants. In Arabidopsis, ethylene was found to affect the ABA/GA balance and involves in breaking dormancy and promoting germination. A recent study showed that ethylene improved the germination of dormant Arabidopsis seeds in dark at 25 °C with a strong decline of ABA/GA ratio after imbibition, whereas a mutant named *ptr6* was insensitive to ethylene-induced germination and the ABA/GA ratio was consequently less affected [46]. Consistently, a greater decrease of ABA/GA ratio was detected in *ereb92* mutant seeds after imbibition (Fig 3C and S6 Fig), we hence propose that the action of ethylene on ZmEREB92-mediated germination might also involves the ABA/GA balance (Fig 8). This may explain why ABA or PAC treatment still partially eliminated the germination difference between *ereb92* mutants and KN5585 (Fig 3A and 3B and S5 Fig).

As key regulators of ethylene signaling, EIN3/EIL transcription factors are involved in numerous ethylene-mediated developmental processes and stress responses [47,48]. It is known that EIN3/EIL members are not completely functional redundant in ethylene signaling [49]. Maize contains nine EIN3/EIL members and ZmEIL7 belongs to the C clade, whose members are found only in angiosperms, suggesting a later appearance in evolution and the involvement in more specific ethylene response [49,50]. The orthologs of *ZmEIL7* in Arabidopsis and rice are *AtEIL4* and *OsEIL6*, respectively, among which *AtEIL4* was found to exhibit restricted expression in embryo [51], but the exact function of both genes has not been characterized yet. Our study identified *ZmEIL7* as a direct target repressed by ZmEREB92 (Fig 4). Further identification of downstream components of ZmEIL7 can be helpful to extend the understanding of the ethylene action during maize seed germination. EIN3/EIL members generally function upstream of ERF transcription factors to regulate ethylene responses [52,53], but here we reveal that *ZmEIL7* is a downstream target of ZmEREB92, presumably reflecting a feedback regulation of ERFs on ethylene signaling.

Another important aspect that affects seed germination is starch mobilization, which provides most growth energy and is mainly mediated by amylases [8]. A role of α-amylase in contributing to the enhanced germination in *ereb92* mutant was supported by the comparable germination using the separated embryo of all genotypes and the increased α-amylase activity in imbibed mutant seeds (Fig 5A–5C). Such increment might be a result of up-regulation of *ZmAMYa2*, which is another direct target suppressed by ZmEREB92 (Fig 5D–5F). The ortholog of *ZmAMYa2* in rice is *RAmy3D*, which encodes an α-amylase that positively control seed germination in rice [14]. Although α-amylase genes are generally considered to be regulated by GA signaling [54], a recent study in rice demonstrated that *RAmy3D* can be directly activated by OsBZR1 and involved in BR signaling-mediated seed germination [14]. Our RNA-seq data showed that *ZmBZR2* and *ZmBZR7* were up-regulated in *ereb92* mutant seeds at 6 HAI compared to KN5585 (Fig 2E), in which *ZmBZR7 is* the orthologous gene of *OsBZR1*. Thus, we hypothesize that the regulation of *ZmAMYa2* by ZmEREB92 might has some crosstalk with BR signaling in maize seed germination.

To ensure successful establishment, plants precisely select the timing for seed germination, in which temperature is an important environmental cue perceived by plant seeds [55]. Maize seeds are hard to germinate in cold condition since it is originated from tropical regions. We found that ZmEREB92 is also a negative regulator in seed germination under cold stress, but interestingly, its expression could also be induced by cold during germination (Fig 7). Therefore, ZmEREB92 is likely to act as an important regulator for the temperature-sensitivity of maize seed that determine the time to germinate. Inhibited expression of *ZmEREB92* in suitable condition ensures timely germination, while induced expression of *ZmEREB92* by cold in turn suppresses the seed germination and leads to cold avoidance behaviors (Fig 8). Such functions appear to be conserved in rice exerted by *OsERF74*, the ortholog of *ZmEREB92* in rice, which could also be up-regulated by cold in previous transcriptomic studies [43]. Recently, AtERF4, which is closely related to ZmEREB92 (Fig 6B), was also identified as a fate switch that negatively regulates the seed state transition from dormancy to germination in Arabidopsis [56], indicating that this clade of ERF members might have conserved functions in both monocots and dicots.

Synchronous seed germination is one of the trait convergently selected in many crops during domestication, meanwhile, it is also required for crop to adapt to different geographical distributions [27,57]. Both *ZmEREB92* and *OsERF74* loci appear to undergo selective sweeps in promoter regions with reduced nucleotide diversity in cultivars compared to wild relatives (Fig 6E and 6F). Correspondingly, *ZmEREB92* displayed lower expression level in maize than teosinte (S15A Fig), suggesting that *ZmEREB92* might have undergone expression divergence during domestication. As the negative regulator of seed germination, the reduced expression of *ZmEREB92* is relevant to the knowledge that the domesticated crops always exhibit enhanced seed germination [27]. The selective evidence for *OsERF74* was only detected in *japonica*, while *indica* displayed an even higher diversity (Fig 6E), which is consistent with previous study [34]. Also, the reduced expression for OsERF74 was found in *temperate japonica* (S15C Fig). Rice *Seed Dormancy* 6 (*SD6*), regulating seed dormancy in a temperature-dependent manner, was found to display lower nucleotide diversity in *japonica* than *indica* [58], which is similar to *OsERF74* (Fig 6E). Interestingly, the expression of *OsERF74* was lower in *japonica* compared with *indica* (S15D Fig). As two major subspecies of cultivated rice domesticated from *rufipogon*, the *indica* cultivars are usually distributed in tropical regions while the *japonica* cultivars are mainly grown in temperate regions [40]. Such altered expression pattern of *OsERF74* is similar with the expression divergence of *ZmEREB92* in temperate and tropical maize (S15B Fig). Given a relatively lower temperature in temperate regions at sowing dates, the decreased expression of *ZmEREB92* and *OsERF74* might be a local adaptation for temperate genotypes to improve the resilience to suboptimal temperature during seed germination.

Interestingly, *OsERF74* exhibited highest expression in floret organs during reproductive process, and could also be induced by cold in anthers [43]. Meanwhile, ZmEREB92 was located within a QTL for days to anthesis in maize using a maize and teosinte (*Zea diploperennis*) recombinant inbred line populations [59]. Although *ZmEREB92* was skipped in a recent study revealing the adaptive variation in *Zea* genus, its downstream target, *ZmEIL7*, was mapped to a region underwent selective sweeps between temperate and tropical maize on Chromosome 2 [60]. These evidences imply that the orthologous gene pair *ZmEREB92/OsERF74* might be related to the adaptation for maize and rice to changing cultivation areas. Exploring the relevant genetic variants and illustrating the biological functions with specific mechanisms in the future will be of great significance.

## Materials and methods

### Plant materials and growth conditions

The CRISPR-Cas9 mutants of *ZmEREB92* were generated from our previous study in KN5585 background [30]. The inbred line Mo17 was also used for germination assay in our study. All these lines were planted in the experimental fields of Sichuan Agricultural University in Chengdu, Sanya and Xishuangbanna at normal condition and seeds were harvested at 45–60 d after artificial self-pollination. The plant height (PH), ear position height (EPH), spike leaf length (SLL) and width (SLW) were measured at milk-ripening stage and the yield-related traits including kernel row number (KNR), kernel number per row (KNPR), ear length (EL), ear diameter (ED), corncob diameter (CD), ear weight (EW), corncob weight (CW) and hundred grain weight (HGW) were measured using the well-pollinated ears. A total of 10 plants were detected for each line as biological replicates. For seedling growth, the seeds were planted in a same pot and grown at the growth chamber at 28 °C/24 °C (day/night) and 16 h light/8 h dark photoperiod with constant irrigation. Three-leaf-stage seedlings were used for cold treatment at 4°C for 4 d with at least three biological replicates.

### Germination assay

Seeds of different genotypes were sterilized with 10% NaClO solution for 20 min and washed 5 times with sterile water. After that, 30 seeds were evenly placed on the filter paper infiltrated with sterile water in the germination box and putted into growth chamber at 28°C with dark condition. Equal amount of sterile water was added every 24 h to keep the filter paper moist. To study the hormone effects on seed germination, water was replaced with 200 mg/L 1-Methylcyclopropene (1-MCP, Fresh Doctor, inhibitor of ET receptor), 100 mg/L Paclobutrazol (PAC, Solarbio, inhibitor of GA biosynthesis), 50 μM ABA (Sigma) or 50 μM ethephon (ETH, Solarbio). For cold and osmotic stress, the sterilized seeds were germinated under 12°C or with 13.5% PEG6000 treatment in dark condition, respectively. The radicle emergence was defined for germination and the number of germinated seeds was recorded every 24 h. Each assay was performed with at least 3 biological replicates. To investigate the internal seed structure during imbibition, the seeds at 0, 6, 24 and 36 h after imbibition (HAI) were cut longitudinally and observed under a stereomicroscope (Olympus-SZX10). The embryo proportion and pericarp thickness were calculated using the ImageJ software with 10 biological replicates.

### Histological sectioning and cytological analysis

To observe the cell size and cell number, the embryos were isolated from the seeds of KN5585 and *ereb92-6* at 36 HAI and then fixed in the FAA buffer (formaldehyde:acetic acid:ethanol:water = 5:5:63:27, V/V/V/V) for 36 h. The samples were sequentially dehydrated with a gradient ethanol, cleared in xylene series, embedded in paraffin and sectioned at 4 μm thickness. The sections were then rehydrated in the gradient xylene and ethanol series and washed with water. After stained with 0.1% toluidine blue for 5 min, the sections were observed and photographed under a light microscope (Leica, DM500). The epidermal cell numbers in radicle and plumule were counted with 5 biological replicates and cell size was calculated with the ImageJ software from 50 cells in 10 randomly selected views from three different sections of each line.

### Water absorption rate

The seeds of KN5585 and *ereb92* mutants were evenly placed on the same filter paper infiltrated with sterile water. The incipient weight of each seed (M_0h_) was measured before imbibition. After 24 h imbibition, the weight of each seed (M_24h_) was measured again. The relative water absorption rate of each line was calculated with 16 biological replicates using the formula: (M_24h_-M_0h_)/M_0h_×100%.

### Measurement of endogenous hormone contents

The seeds of KN5585 and *ereb92-6* at 0 and 36 HAI were collected, grounded into powder with liquid N_2_ and extracted with 1 mL methanol/water/formic acid (15:4:1, V/V/V). After centrifugation for 5 min at 4 °C, 1200 rpm, the extracts were concentrated and re-dissolved with 100 μL 80% methanol/water (V/V) solution for LC-MS/MS analysis. The endogenous GAs, ABA and ACC contents were detected by MetWare (http://www.metware.cn/) based on the AB SciexQTRAP 6500 LC-MS/MS platform. Three biological replicates from each line were analyzed.

### RNA extraction and qRT-PCR

To determine the expression pattern during seed germination, the seeds of different genotypes were imbibed for 2, 6, 12, 24 and 36 h with or without a certain treatment and grounded into a fine powder in liquid N_2_. The RNA was extracted with TRNzol reagents (Tiangen) according to the manufacturer’s instructions and subsequently 1 μg of RNA was used for cDNA synthesis with the reverse transcriptase (Vazyme). The quantitative real-time RT-PCR (qRT-PCR) was executed on a StepOne Plus Real-Time PCR System (Applied Biosystems, ABI) using the SYBR Green Master Mix (Takara). All expression levels were calculated by the ΔΔCT method using the elongation factor *Ef1a* as the reference gene [61]. All experiments were performed with at least three biological replicates. Primers were listed in S5 Table.

### RNA-seq analysis

Total RNA was extracted from the of KN5585 and *ereb92-6* at 0 and 6 HAI seeds with three biological replicates and RNA integrity was evaluated with Agilent 2100 bioanalyzer. The RNA libraries were prepared and sequenced by Novogene (Beijing) on an Illumina Novaseq platform. Raw data was subjected to FastQC software for quality control and then the clean reads were aligned to maize genome (B73 RefGen_V4, AGPv4) using Hisat2 v2.0.5. FPKM, which was generated by counting the reads numbers mapped to each gene by featureCounts v1.5.0-p3, and used to estimate gene expression levels. Differential expression analysis was performed using the DESeq2 R package (1.20.0) and defined with a corrected *P*-value<0.05 (Benjamini and Hochberg’s approach) and absolute foldchange (FC) ≥2. Differentially expressed genes (DEGs) were further implemented by clusterProfiler R package for Gene Ontology (GO) and Kyoto Encyclopedia of Genes and Genomes (KEGG) enrichment analysis. The TBtools software was used to display the expression of DEGs by heatmaps.

### *Cis*-element enrichment analysis

The upstream 1500 bp of the gene was extracted as the promoter sequence. The *cis*-elements were predicted using the Plant Transcriptional Regulatory Map (http://plantregmap.gao-lab.org/binding_site_prediction.php) with the *P* value < 10^−5^ as the threshold for filtering. The retrieved *cis*-elements were indicated as transcription factor binding sites (TFBS) and then the percentage of genes with each TFBS in promoter was calculated and compared. Genes that were not differentially expressed in any comparison were analyzed as the control. The bootstrap method was used to test the significant enrichment.

### Phylogenetic analysis

A literature search or database search based on PhyloGenes website (http://www.phylogenes.org/) was performed to identify the group VIII subfamily ERF transcription factors, EIN3/EIL members and α-amylases in *Zea mays* (RefGen_V4), *Oryza sativa* (*Japonica Group* IRGSP-1.0) and *Arabidopsis thaliana* (TAIR10), and the sequences were downloaded from MaizeGDB (https://www.maizegdb.org/) and *EnsemblPlants* (http://plants.ensembl.org/index.html). Amino acid sequences were aligned in MEGA software with ClustalW method using the maximum likelihood method. The iTOL online website (https://itol.embl.de/) was used to visualize the tree. Accession numbers for sequence data used in phylogenetic analysis can be found in S6 Table.

### Dual-luciferase reporter assay

The promoter sequences of downstream genes were downloaded from *Phytozome* v13.0 (https://phytozome-next.jgi.doe.gov/) based on *Zea mays RefGen_V4*. The promoters were cloned from the genomic DNA of B73 inbred line and then constructed into the reporter vector pGreenII 0800-LUC. The coding sequence of *ZmEREB92* was cloned into the effecter vector pGreenII 62-SK driven by the 35S promoter. The promoter-LUC plasmids were co-transformed with *ZmEREB92* into maize protoplasts via PEG-mediated transfection method as described previously and REN was used as the internal control [62]. After incubated for 16h, total proteins were extracted from the transfected cells with the dual-luciferase assay reagents (Promega) and analyzed on a luminometer (Thermo Scientific Varioskan LUX). The LUC/REN ratio was used to defined the promoter activity. At least four biological replicates were performed for each experiment.

### Yeast one-hybrid (Y1H) assay

To test the direct binding of ZmEREB92 to *ZmEIL7* promoters, the yeast one-hybrid assays were carried out based on the GAL4-AbA system (Clontech) [63]. The GAL4-activating domain fused ZmEREB92 (AD-EREB92) was obtained in our previous study and further mutation of two EAR motifs was performed to generate AD-EREB92(ΔEAR1/2). Promoter region of *ZmEIL7* was cloned into the pAbAi vector and then linearized at *Bbs*I or *BstB*I site before transformed into the Y1H gold strain. Subsequently, the AD-EREB92 or AD-EREB92(ΔEAR1/2) was transformed into yeast cells integrated with p*ZmEIL7*-AbAi vector and the transformed cells were grown on the selective medium SD/-Ura/-Leu and SD/-Ura/-Leu/AbA for 2–4 d. p53 and empty pGADT-7 (AD) were also transformed as the negative control.

### Electrophoretic mobility shift assay (EMSA)

The CDS of ZmEREB92 was connected into pET32a with a His-tag fused to the C terminus and then transformed into BL21 competent cells. The recombinant protein was induced by 0.5 mM IPTG and purified with Ni-NTA beads (Smart-Lifesciences). The promoter fragments of *ZmEIL7* and *ZmAMYa2* were labeled with biotin (Sango). Afterwards, 0.2 pmol labeled probes were incubated with equal amount of ZmEREB92-His protein for 30 min at 28 °C. The unlabeled probes were used as cold competitors and added with 200× amount for competition. The mixtures were further separated in 6.5% native page gel and transferred to the 0.45μm Nylon membrane (Sango). The probes were detected with a chemiluminescence EMSA kit (Beyotime). Probes were listed in S5 Table.

### Analysis of α-amylase activity

The seeds of KN5585 and *ereb92* mutants imbibed for 24 h were used to quantified the α-amylase activity with a micro α-amylase assay kit (Solarbio). Briefly, the imbibed seeds were ground to a fine powder in liquid N_2_ and 0.8 mL ddH_2_O was added into 0.1 g powder and mixed thoroughly. After 15 min incubation at room temperature, the supernatant was collected by centrifuging at 6000 g for 10 min and further used for the detection of α-amylase activity. Three biological replicates from each line were analyzed.

### Bioinformatic analysis

Sequence alignments was performed with ClustalW method and the phylogenetic tree was constructed with the Maximum- Likelihood method using the MEGA software. All phylogenetic trees were visualized through iTOL online website (https://itol.embl.de/). The orthologous genes were identified using the public database including maizeGDB (https://www.maizegdb.org/), *EnsemblPlants* (http://plants.ensembl.org/index.html) and Gramene Maize (https://maize-pangenome.gramene.org/). The genomic sequences and annotations of maize chromosome 8 and rice chromosome 5 were downloaded from *EnsemblePlants* database based on *Zm-B73-REFERENCE-NAM-5*.*0* and *Oryza sativa Japonica* Group IRGSP-1.0, respectively. Then the syntenic analysis between maize chromosome 8 and rice chromosome 5 was performed with TBtools [64]. The constitutive expression patterns of *ZmEREB92* and *OsERF74* from qTeller dataset (http://qteller.maizegdb.org) and Rice Expression Profile Database (RiceXPro, https://ricexpro.dna.affrc.go.jp/) were shown in heatmap drawn by TBtools [64–66].

The expression profiles of *ZmEREB92* and *OsERF74* in different species were derived from several public high-throughput RNA-seq data. The dataset used for the expression analysis of *ZmEREB92* in wild and cultivated maize is deposited in GEO database under the accession number GSE30036, which sequenced the above-ground tissue of 38 maize genotypes and 24 teosinte genotypes [35]. We extracted the expression value of *ZmEREB92* based on the probe ID CHR08RS119567227, CHR08RS119567281 and CHR08RS119568280. The transcriptomes for temperate maize and tropical maize cultivars are retrieved from the GenBank Sequence Read Archive (SRA) under the accession code SRP026161, which performed on the immature seeds of 15 days after pollination for these 368 lines [36]. The RPKM of *ZmEREB92* in SRP026161 is normalized for analysis. The expression data for *OsERF74* is collected from the rice library in Plant Public RNA-seq Database (http://ipf.sustech.edu.cn/pub/plantrna/) by searching the gene ID *LOC_Os05g41780* and BioProject ID PRJNA428294, PRJNA597070 and PRJNA292458, in which PRJNA428294 performed on the panicles of wild and cultivated rice, PRJNA597070 performed on the young leaf of different cultivated rice accessions and PRJNA292458 performed on the seedlings of six accessions from *indica* and *japonica* [37–39,67].

### Nucleotide diversity analysis

Since ZmEREB92 was not assembled in *B73_RefGen V4* genome, a small panel of genomic sequences across *ZmEREB92* locus from 26 maize accessions and 8 teosinte accessions were downloaded from maizeGDB based on Zm-B73-REFERENCE-NAM-5.0 reference genome. Genomic sequence across *OsERF74* locus using the data from Rice Super Pan-genome Information Resource Database (RiceSuperPIRdb, http://www.ricesuperpir.com/), including 132 *indica* accessions, 57 *japonica* accessions and 23 *rufipogon* accessions [68]. Nucleotide diversity was calculated under a 50-bp sliding window and 25 bp step size for maize/teosinte comparison or a 100-bp sliding window and 25 bp step size for *O*. *sativa/O*. *rufipogon* comparison using DnaSP 5.0 software (http://www.ub.edu/dnasp/) [69].

### Generation of rice *Oserf74* knockout lines for seed germination assays

To generate the CRISPR/Cas9-mediated *OsERF74* knockout mutation in rice, the sequence 5’-GGCACCGGCCAGCACTTCCGTGG-3’ from 207 to 226 bp of *OsERF74* CDS was selected as the guideRNA and introduced into the pCBSG032 CRISPR/Cas9 vector. Then the construct was transformed into rice callus with the *Agrobacterium tumefaciens* strain EHA105. The positive transformants were identified by hygromycin soaking and sequencing confirmation. Two T_2_ knockout lines were obtained for germination assays as described above for maize seeds. The difference is that the germination of rice seeds is at 25 °C for normal condition and 15 °C for cold condition.

### Chilling treatment at seedling stage

The three-leaf-stage seedlings of KN5585 and two *ereb92* mutant lines were used for chilling treatment. The seedlings were transferred to the growth chamber at 4°C with a 16 h light/8 h dark photoperiod for 4 days and then recovered at 25°C for 2 days. After recovery, the photographs were taken and the survival rates were calculated. As described previously, the relative injured area (%) was defined with the ratio of the wilting area to the whole area of the second leaves using the Image J software [70]. Each experiment was performed with at least three biological replicates.

### Statistical analysis

The statistical analysis was carried out using the GraphPad Prism 8.0 software. Briefly, multiple comparison was performed using one-way or two-way ANOVA followed by Tukey tests or LSD tests and differences between two data sets were calculated by the two-side Student’s *t* test or Mann-Whitney U test.

## Supporting information

S1 Fig The seed germination of KN5585 and *ereb92* mutants harvested from different province and year in China. The seed germination rates at the 3DAI of KN5585 and *ereb92* mutant harvested from Xishuangbanna (Yunnan) at 2019, Chengdu (Sichuan) at 2020 and Sanya (Hainan) at 2021. Error bars indicate mean ± SE (n = 3). n. s. indicates no significant difference (one-way ANOVA followed by Turkey tests, *P*>0.05).
(TIF)

S2 Fig Loss-of-function of *ZmEREB92* didn’t affect the pericarp thickness and water absorption in maize seed. A. The longitudinal section at 24 HAI showing the pericarp of KN5585 and ereb92 mutants. B-C. Box plots represent the distribution of the pericarp thickness (B) and water absorption rate (C) of the seeds of KN5585 and *ereb92* mutants. The bars indicate the median, and the lower and upper quartiles. The circles represent for individual datapoints of biological replicates in each line. n. s. indicates no significant difference (one-way ANOVA followed by Turkey tests, *P*>0.05).
(TIF)

S3 Fig Transcriptome analysis for the DEGs in KN5585 and *ereb92* mutant seeds after imbibition. A-B. GO (A) and KEGG (B) analysis of the of the DEGs in the comparison of *ereb92*_6h vs KN5585_6h. C. The heatmap shows Log_2_FC of selected DEGs in the comparison groups of *ereb92*_6h vs *ereb92*_0h or *ereb92*_6h vs KN5585_6h.
(TIF)

S4 Fig *Cis*-enrichment analysis for the promoters of DEGs. A. The Venn diagram shows the number of overlapped DEGs in the comparison groups of KN5585_6h vs KN5585_0h (S1) and *ereb92*_6h vs *ereb92*_0h (S2). B-G. The percentage of DEGs with each promoter-contained TFBS in different comparison groups. The error bar represents the standard deviation by bootstrap test.
(TIFF)

S5 Fig Comparison of the *P* value reveals the difference of effects of each treatment on germination between KN5585 and *ereb92* mutants. The *P* values were calculated from the date regarding to Fig 3B by student *t*-test.
(TIF)

S6 Fig The ABA/GA ratio of KN5585 and *ereb92*-6 at 0 and 36 HAI. The ratio was calculated from the data regarding to Fig 3C. Error bars indicate mean ± SE (n = 3). Different lowercases represent significant difference (one-way ANOVA followed by Turkey tests, *P*<0.05).
(TIF)

S7 Fig Ethylene signaling positively regulate seed germination in maize Mo17 inbred line. A. Germination performance at the 2HAI of Mo17 seeds under normal condition (CK), 50 μM ethephon (ETH) and 200 mg/L 1-MCP treatment. B. Time course germination from 1–4 DAI for Mo17 seeds under different treatments. Error bars indicate mean ± SE (n = 3). C. The longitudinal section of Mo17 seeds at 0 and 36 HAI under different treatments. Embryo region was sketched with yellow dash line. D. The percentage of embryo for Mo17 seeds at 0 and 36 HAI under different treatments. The embryo proportion is calculated by ImageJ software. The circles are represented for individual datapoints of biological replicates in each line. Error bars indicate mean ± SE (n = 8). Statistical significance was determined individually for 0 HAI and 36 HAI. Different lowercases represent significant difference (one-way ANOVA followed by Turkey tests, *P*<0.05). E, F. The expression of *ZmEREB92* in Mo17 seeds at 2, 6, 12, 24 and 36 HAI under ETH (E) or 1-MCP treatment (F). Error bars indicate mean ± SE (n = 3). *Ef1a* was used as the reference gene and relative expression level was normalized to one biological replicate of 2 HAI. Asterisks indicate significant difference compared to 2 HAI (one-way ANOVA followed by LSD tests, *P<0.05, **P<0.01).
(TIFF)

S8 Fig Promoter analysis for ethylene signaling related genes. A. The distribution of GCC-boxes in the promoter of four ethylene signaling genes including *ZmEIL7*, *ZmEIL4*, *ZmETR2* and *ZmEBF2*. The GCC-box was indicated with blue sticks. TSS, Transcription start site. B. Schematic of the reporter, effector and empty vector used in the transient DLR assays in maize protoplast.
(TIF)

S9 Fig Phylogenetic analysis of ZmEREB92 downstream targets. Phylogenetic analysis of EIN3/EIL members and α-amylase genes in maize, rice and Arabidopsis. MEGA software was used to perform the sequence alignment with ClustalW method and the phylogenetic trees was constructed with the Maximum-Likelihood method. The iTOL online website (https://itol.embl.de/) was used to visualize the tree.
(TIFF)

S10 Fig The distribution of GCC-boxes in candidate gene promoters. A-C. The identification of GCC-boxes for the promoters of *ZmAMYs* (A), *OsEIL6* and *RAmy3D* (B) and *ZmCESAs* (C). The GCC-box was indicated with blue sticks. TSS, Transcription start site.
(TIFF)

S11 Fig Phylogenetic analysis of ZmEREB92. Phylogenetic analysis of ZmEREB92 with other group VIII ERF family members in maize, rice and Arabidopsis. MEGA software was used to perform the sequence alignment with ClustalW method and the phylogenetic trees was constructed with the Maximum-Likelihood method. The iTOL online website (https://itol.embl.de/) was used to visualize the tree.
(TIF)

S12 Fig The constitutive expression of ZmEREB92 and OsERF74. A-B. The heatmap showing expression profile of ZmEREB92 (A) and OsERF74 (B).
(TIF)

S13 Fig Mutation sites of *Oserf74* knockout lines generated by CRISPR-Cas9. (TIF)

S14 Fig ZmEREB92 has not been assembled in chromosome 8 of B73 RefGen_v4. Comparison of 0.5 Mb genomic region across *ZmEREB92* locus at chromosome 8 between three versions of B73 reference genome. The same genes are connected by dash lines. The numbers reflect different genes. ZmEREB92 is indicated by number 14 with red dash line. Detail information for genes were listed in S4 Table.
(TIF)

S15 Fig The expression profile of *ZmEREB92* and *OsERF74* in different varieties. A. The expression level of *ZmEREB92* in teosinte and cultivated maize. B. The normalized RPKM of *ZmEREB92* in different maize subpopulations. NSS, non-stiff stalk. SS, stiff-stalk. TEM, temperate. TST, tropical/subtropical. C. The expression level of *OsERF74* in wild and cultivated rice. Tro, tropical. Tem, temperate. D. The expression level of *OsERF74* in different rice subspecies. A-D. The values were displayed Tukey box-plot. Number marked for each data is the exact *P* value (Mann-Whiney U test).
(TIFF)

S16 Fig Cold-related genes are largely upregulated by *ZmEREB92* mutation during imbibition. The heatmap shows the log_2_FC of differentially expressed *DREBs* and *CIPKs* in the comparison groups of *ere92*_6h vs *ere92*_0h or *ere92*_6h vs KN5585_6h.
(TIF)

S17 Fig Loss-of-function of *ZmEREB92* improves cold tolerance of maize seedlings. A-C. Chilling phenotype (A), injured area (B) and survival rate (C) of KN5585 and *ereb92* mutants after 4 days treatment under cold condition (4 °C). (G, H) Error bars indicate mean ± SE (n = 6 for G, n = 3 for H). Asterisks indicate significant difference (Student’s *t*-test, **P<0.01).
(TIF)

S18 Fig Loss-of-function of *ZmEREB92* rarely affected the gemination under osmotic stress. A. The germination performance of KN5585 and *ereb92* mutants under 13.5% PEG6000 treatment at 5 DAI. B. The time course germination from 1–7 DAI of KN5585 and *ereb92* mutants under 13.5% PEG6000 treatment. Error bars indicate mean ± SE (n = 3). The expression of *ZmEREB92* in Mo17 seeds under 13.5% PEG6000 treatment at 2, 6, 12, 24 and 36 HAI. Error bars indicate mean ± SE (n = 3). *Ef1a* was used as the reference gene and relative expression level was normalized to one biological replicate of 2 HAI. Asterisks indicate significant difference compared to 2 HAI (one-way ANOVA followed by LSD tests, *P<0.05).
(TIFF)

S1 Table GO enrichment analysis of up-regulated genes. (XLSX)

S2 Table KEGG enrichment analysis of up-regulated genes. (XLSX)

S3 Table List of DEGs analyzed in this study. (XLSX)

S4 Table Genes within 0.5 Mb region across ZmEREB92 locus on chromosome 8 assembled by three versions of B73 reference genome. (XLSX)

S5 Table List of primers used in this study. (XLSX)

S6 Table List of genes used in constructing the phylogenetic trees. (XLSX)

S1 Data The complete RNA-seq results containing the expression level and the annotation of all detected genes. (XLSX)
