# Supplementary figures and images for: ZmEREB92 plays a negative role in seed germination by regulating ethylene signaling and starch mobilization in maize
Source: PLoS Genet. 2023 Nov 17;19(11):e1011052. doi: 10.1371/journal.pgen.1011052 (PMC10691696; doi:10.1371/journal.pgen.1011052)

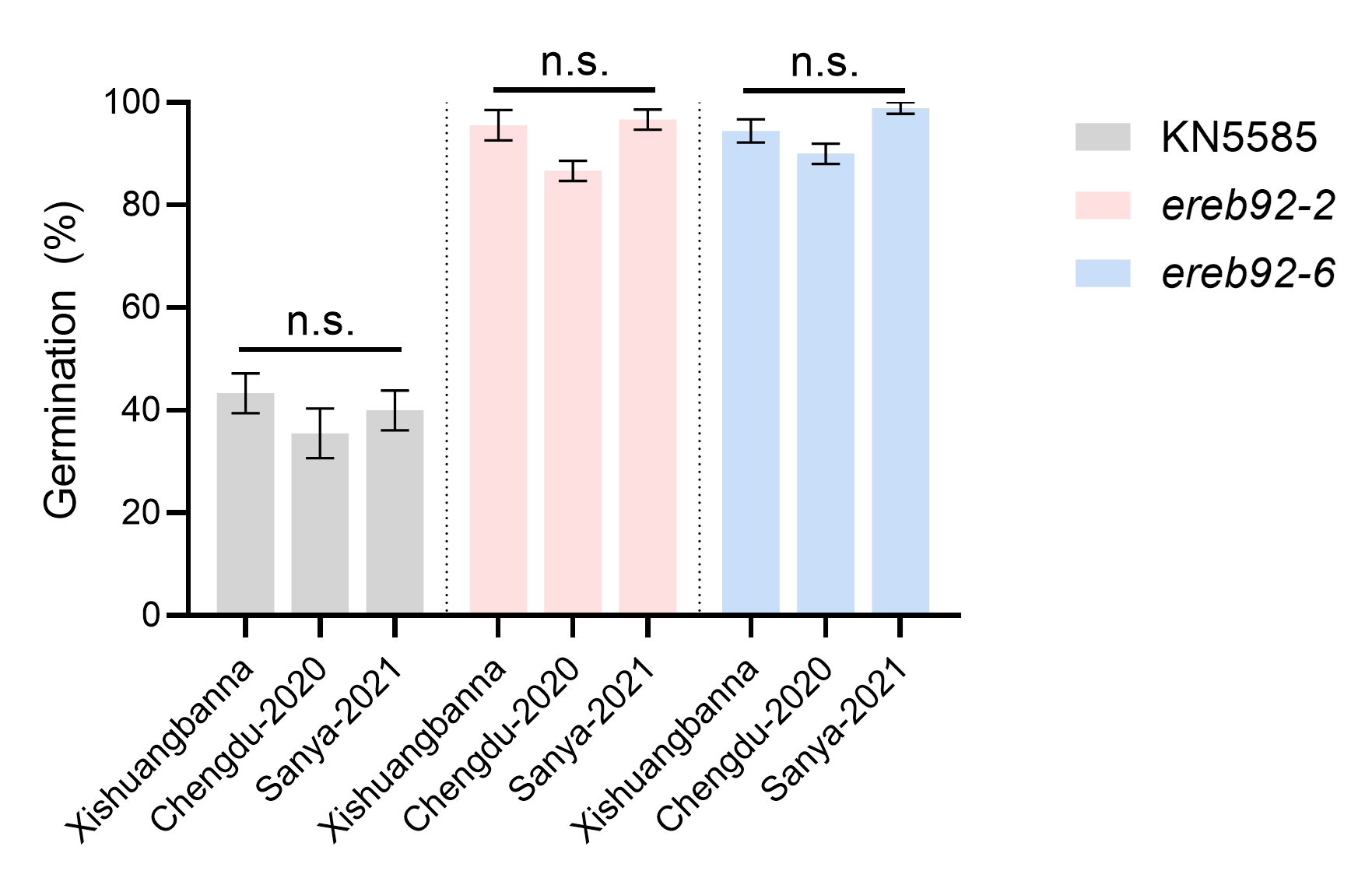

Supplement: S1 Fig — The seed germination rates at the 3DAI of KN5585 and ereb92 mutant harvested from Xishuangbanna (Yunnan) at 2019, Chengdu (Sichuan) at 2020 and Sanya (Hainan) at 2021. Error bars indicate mean ± SE (n = 3). n. s. indicates no significant difference (one-way ANOVA followed by Turkey tests, P>0.05). (TIF) [file pgen.1011052.s001.tif]

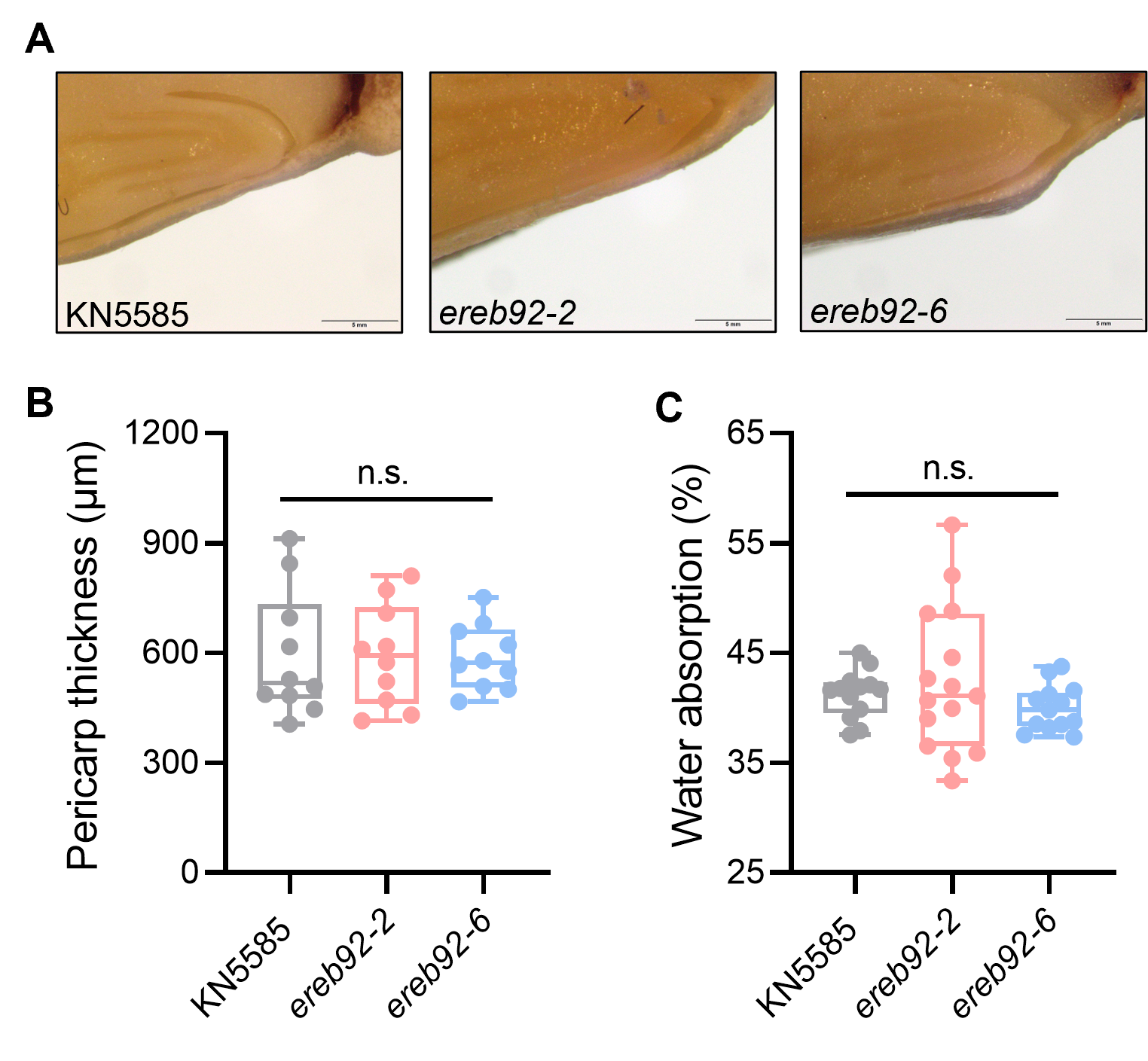

Supplement: S2 Fig — A. The longitudinal section at 24 HAI showing the pericarp of KN5585 and ereb92 mutants. B-C. Box plots represent the distribution of the pericarp thickness (B) and water absorption rate (C) of the seeds of KN5585 and ereb92 mutants. The bars indicate the median, and the lower and upper quartiles. The circles represent for individual datapoints of biological replicates in each line. n. s. indicates no significant difference (one-way ANOVA followed by Turkey tests, P>0.05). (TIF) [file pgen.1011052.s002.tif]

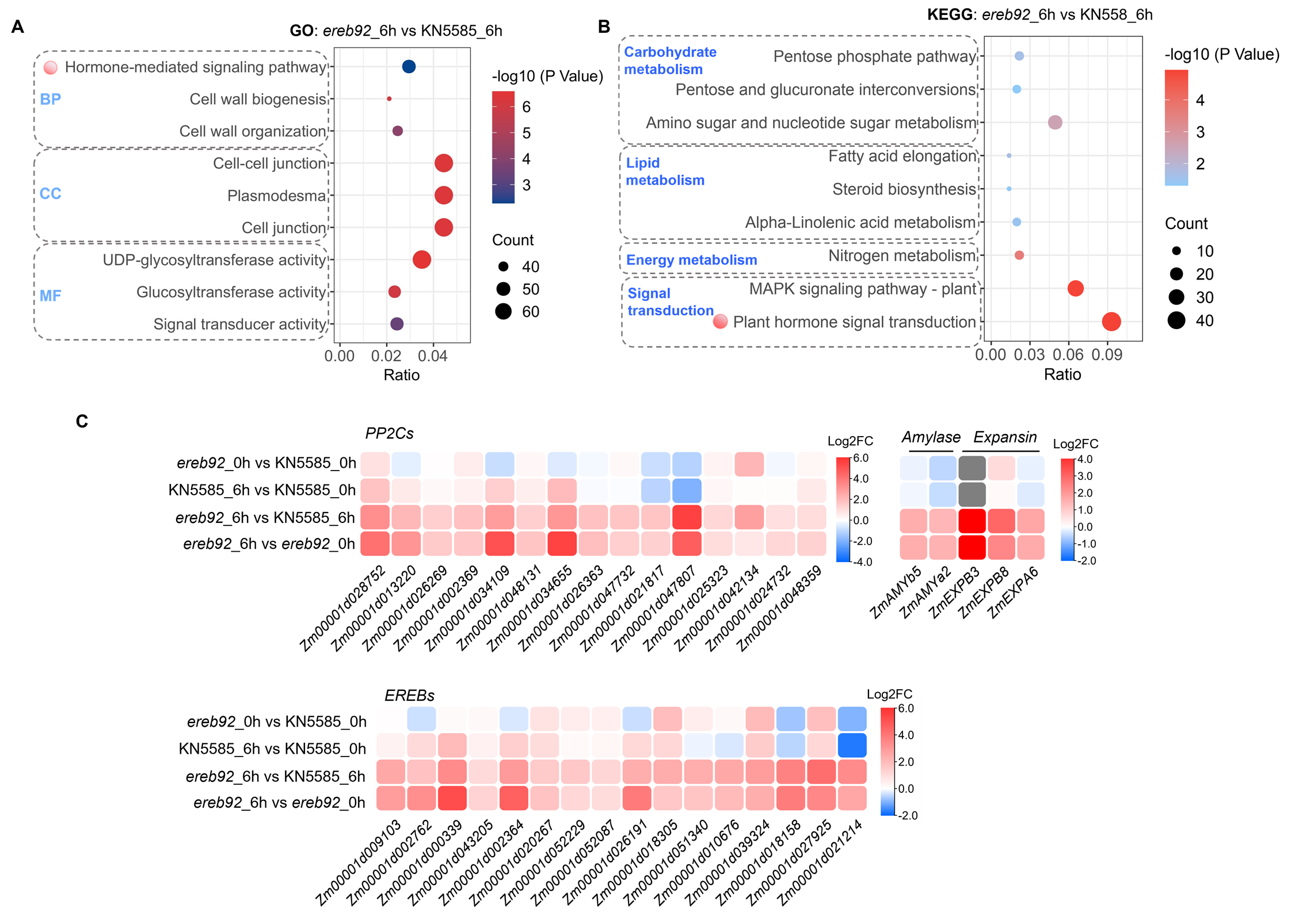

Supplement: S3 Fig — A-B. GO (A) and KEGG (B) analysis of the of the DEGs in the comparison of ereb92_6h vs KN5585_6h. C. The heatmap shows Log2FC of selected DEGs in the comparison groups of ereb92_6h vs ereb92_0h or ereb92_6h vs KN5585_6h. (TIF) [file pgen.1011052.s003.tif]

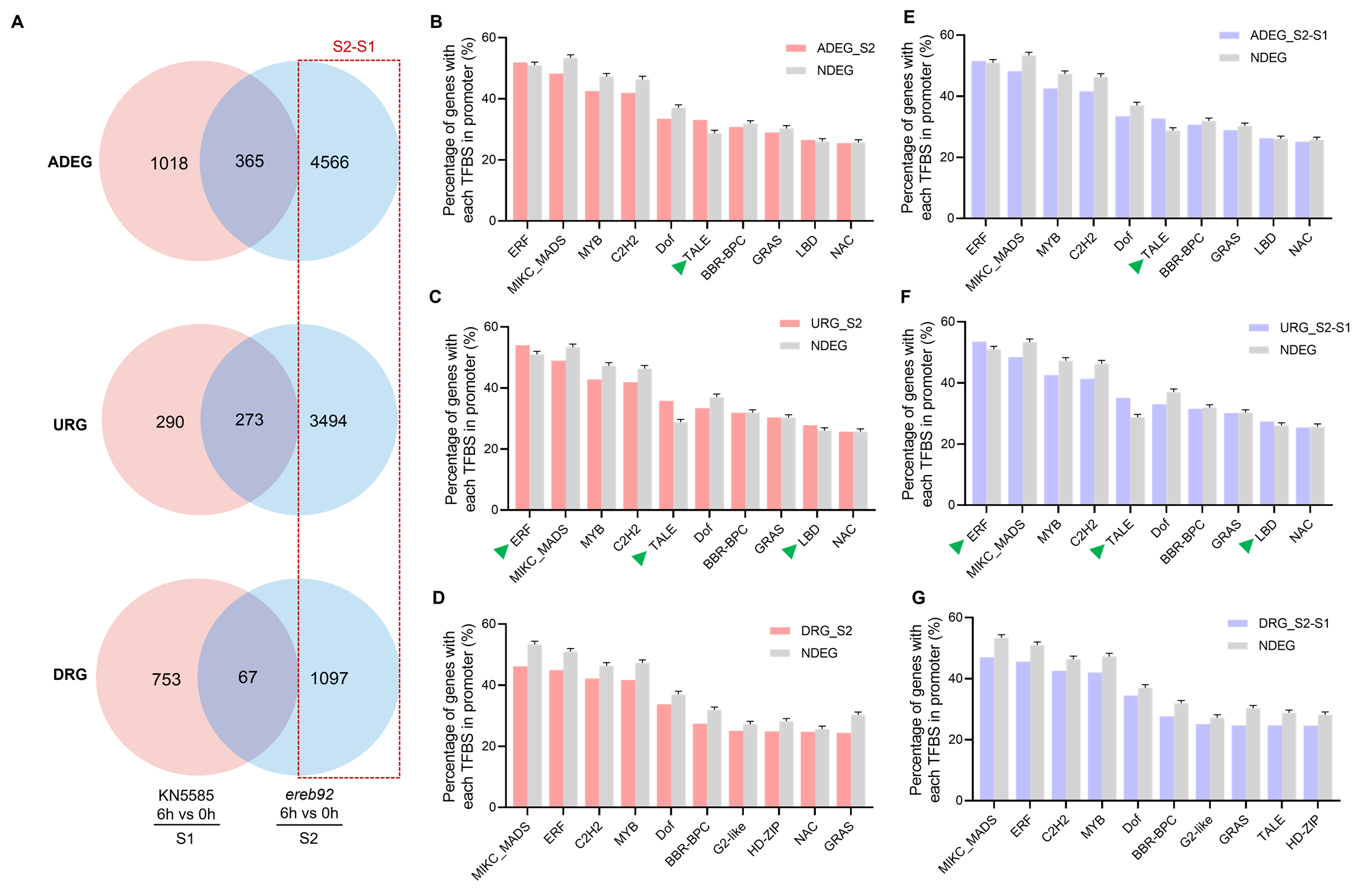

Supplement: S4 Fig — A. The Venn diagram shows the number of overlapped DEGs in the comparison groups of KN5585_6h vs KN5585_0h (S1) and ereb92_6h vs ereb92_0h (S2). B-G. The percentage of DEGs with each promoter-contained TFBS in different comparison groups. The error bar represents the standard deviation by bootstrap test. (TIFF) [file pgen.1011052.s004.tiff]

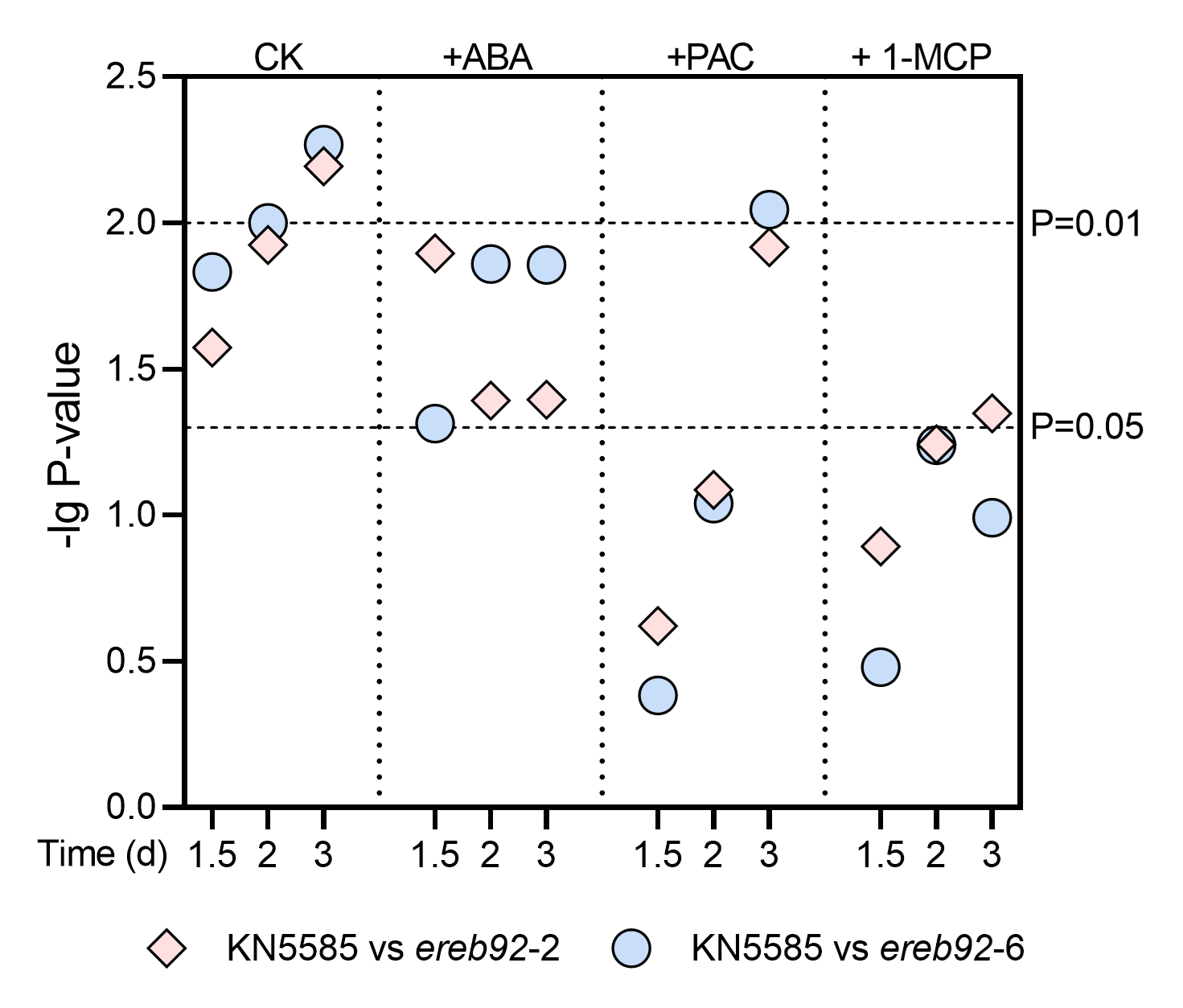

Supplement: S5 Fig — The P values were calculated from the date regarding to Fig 3B by student t-test. (TIF) [file pgen.1011052.s005.tif]

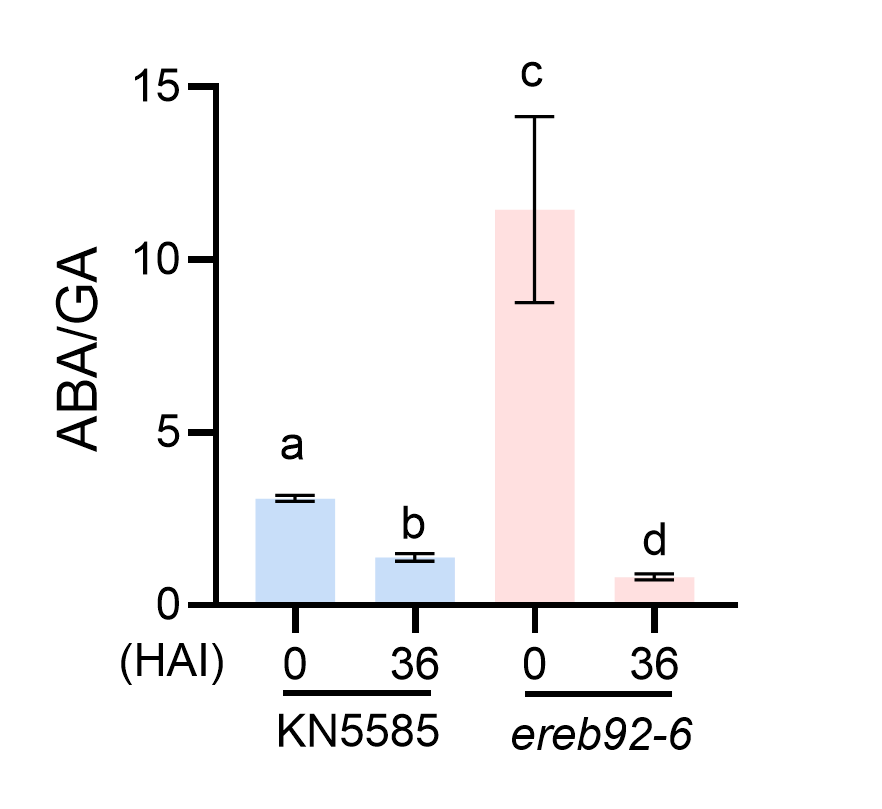

Supplement: S6 Fig — The ratio was calculated from the data regarding to Fig 3C. Error bars indicate mean ± SE (n = 3). Different lowercases represent significant difference (one-way ANOVA followed by Turkey tests, P<0.05). (TIF) [file pgen.1011052.s006.tif]

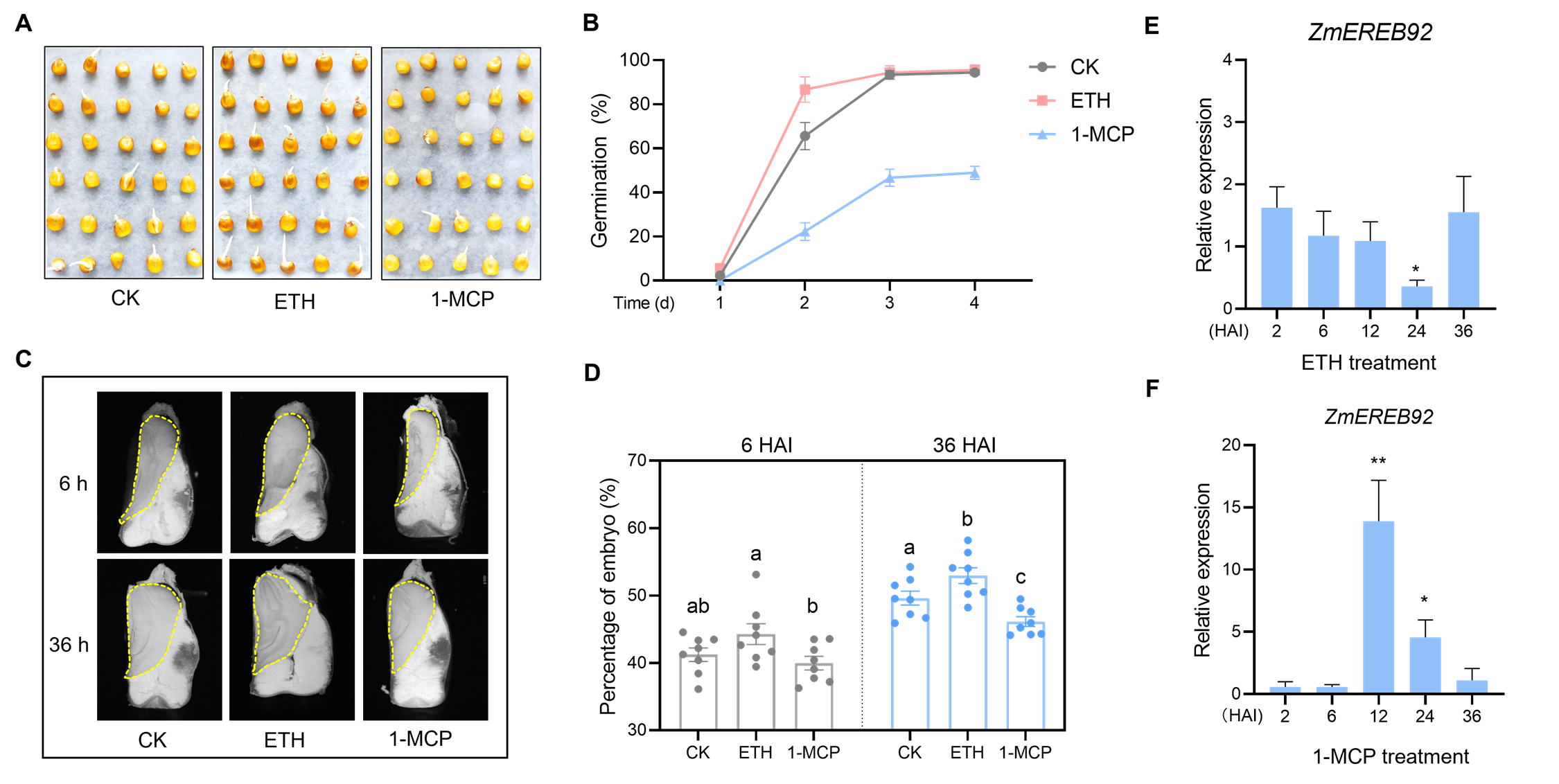

Supplement: S7 Fig — A. Germination performance at the 2HAI of Mo17 seeds under normal condition (CK), 50 μM ethephon (ETH) and 200 mg/L 1-MCP treatment. B. Time course germination from 1–4 DAI for Mo17 seeds under different treatments. Error bars indicate mean ± SE (n = 3). C. The longitudinal section of Mo17 seeds at 0 and 36 HAI under different treatments. Embryo region was sketched with yellow dash line. D. The percentage of embryo for Mo17 seeds at 0 and 36 HAI under different treatments. The embryo proportion is calculated by ImageJ software. The circles are represented for individual datapoints of biological replicates in each line. Error bars indicate mean ± SE (n = 8). Statistical significance was determined individually for 0 HAI and 36 HAI. Different lowercases represent significant difference (one-way ANOVA followed by Turkey tests, P<0.05). E, F. The expression of ZmEREB92 in Mo17 seeds at 2, 6, 12, 24 and 36 HAI under ETH (E) or 1-MCP treatment (F). Error bars indicate mean ± SE (n = 3). Ef1a was used as the reference gene and relative expression level was normalized to one biological replicate of 2 HAI. Asterisks indicate significant difference compared to 2 HAI (one-way ANOVA followed by LSD tests, *P<0.05, **P<0.01). (TIFF) [file pgen.1011052.s007.tiff]

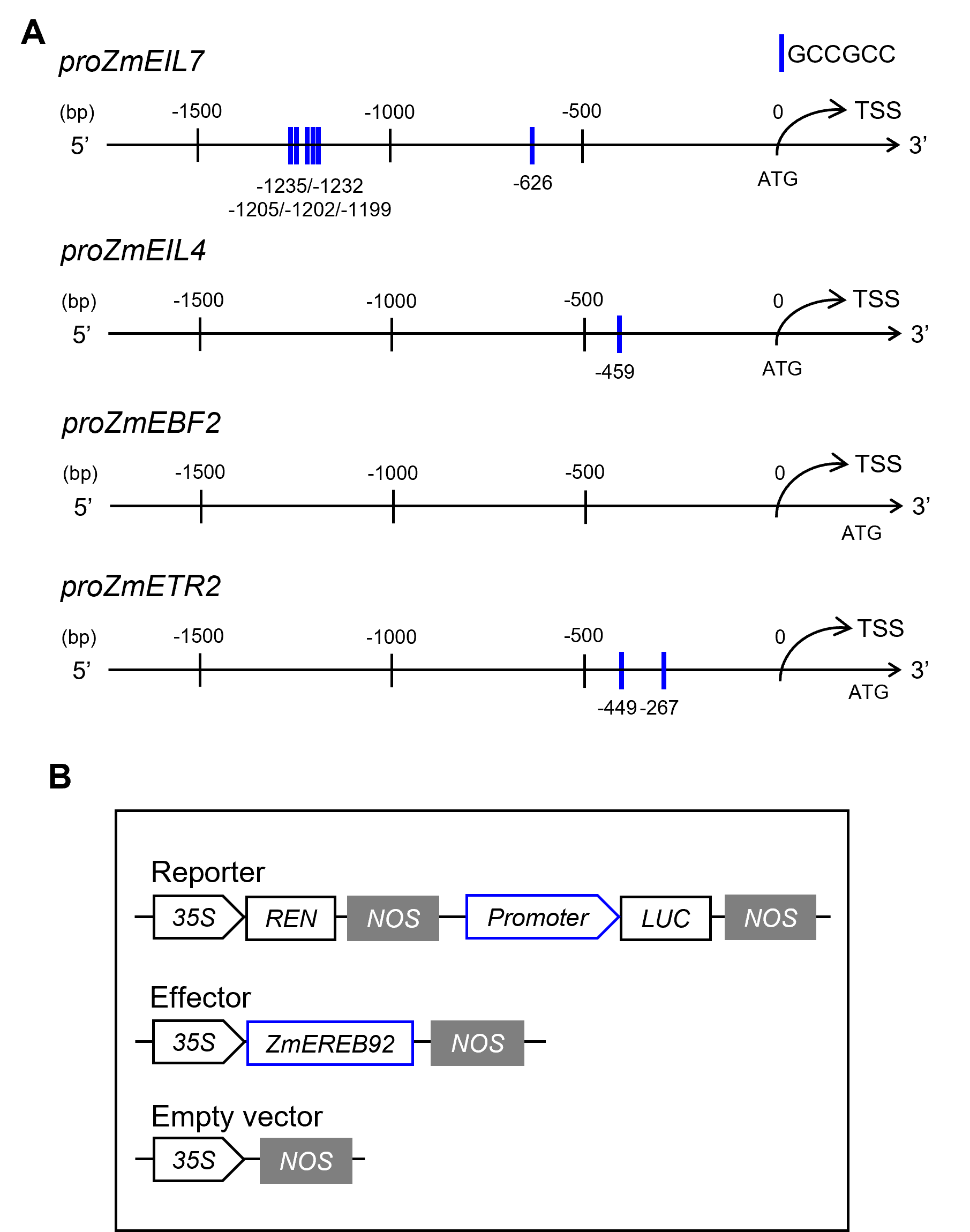

Supplement: S8 Fig — A. The distribution of GCC-boxes in the promoter of four ethylene signaling genes including ZmEIL7, ZmEIL4, ZmETR2 and ZmEBF2. The GCC-box was indicated with blue sticks. TSS, Transcription start site. B. Schematic of the reporter, effector and empty vector used in the transient DLR assays in maize protoplast. (TIF) [file pgen.1011052.s008.tif]

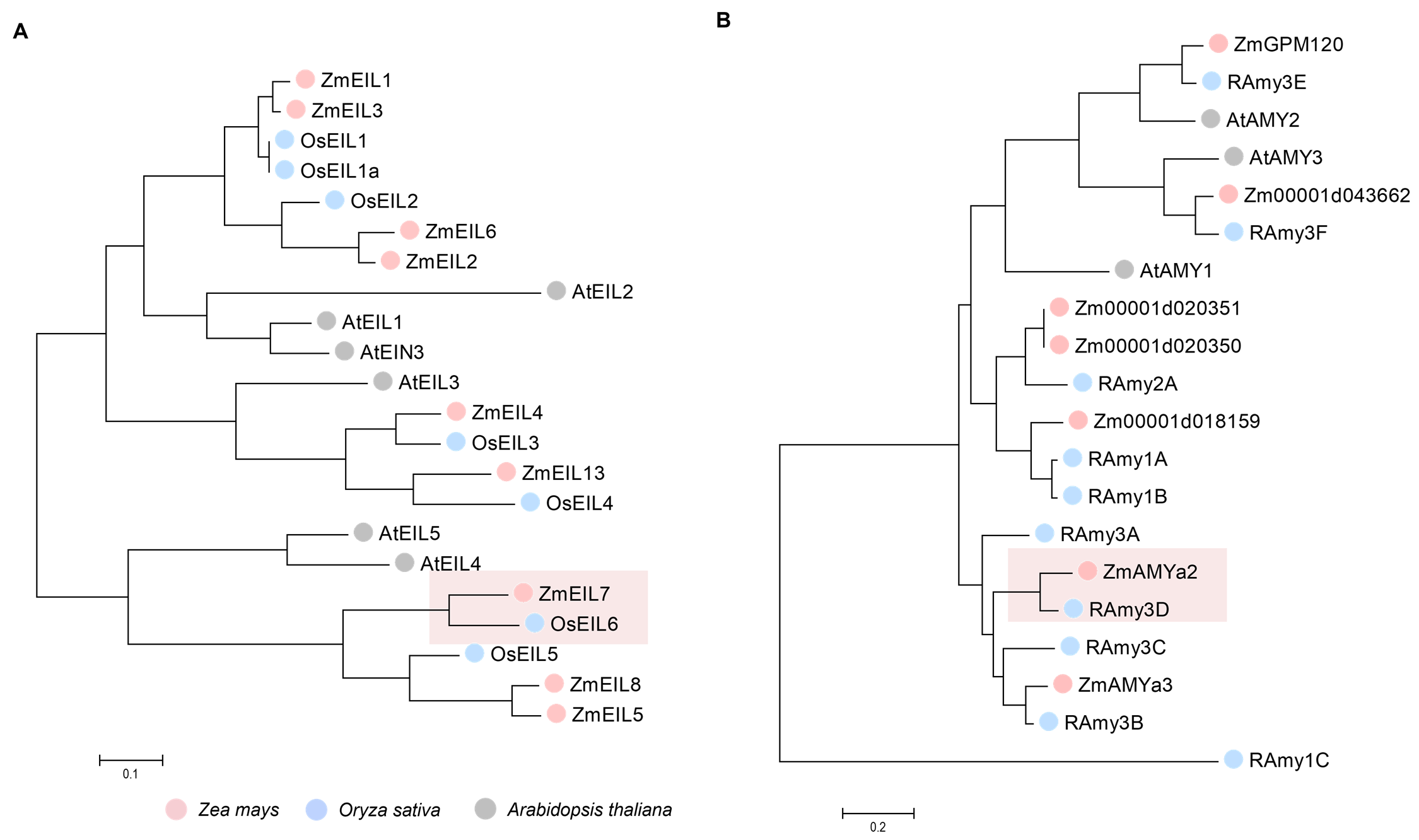

Supplement: S9 Fig — Phylogenetic analysis of EIN3/EIL members and α-amylase genes in maize, rice and Arabidopsis. MEGA software was used to perform the sequence alignment with ClustalW method and the phylogenetic trees was constructed with the Maximum-Likelihood method. The iTOL online website (https://itol.embl.de/) was used to visualize the tree. (TIFF) [file pgen.1011052.s009.tiff]

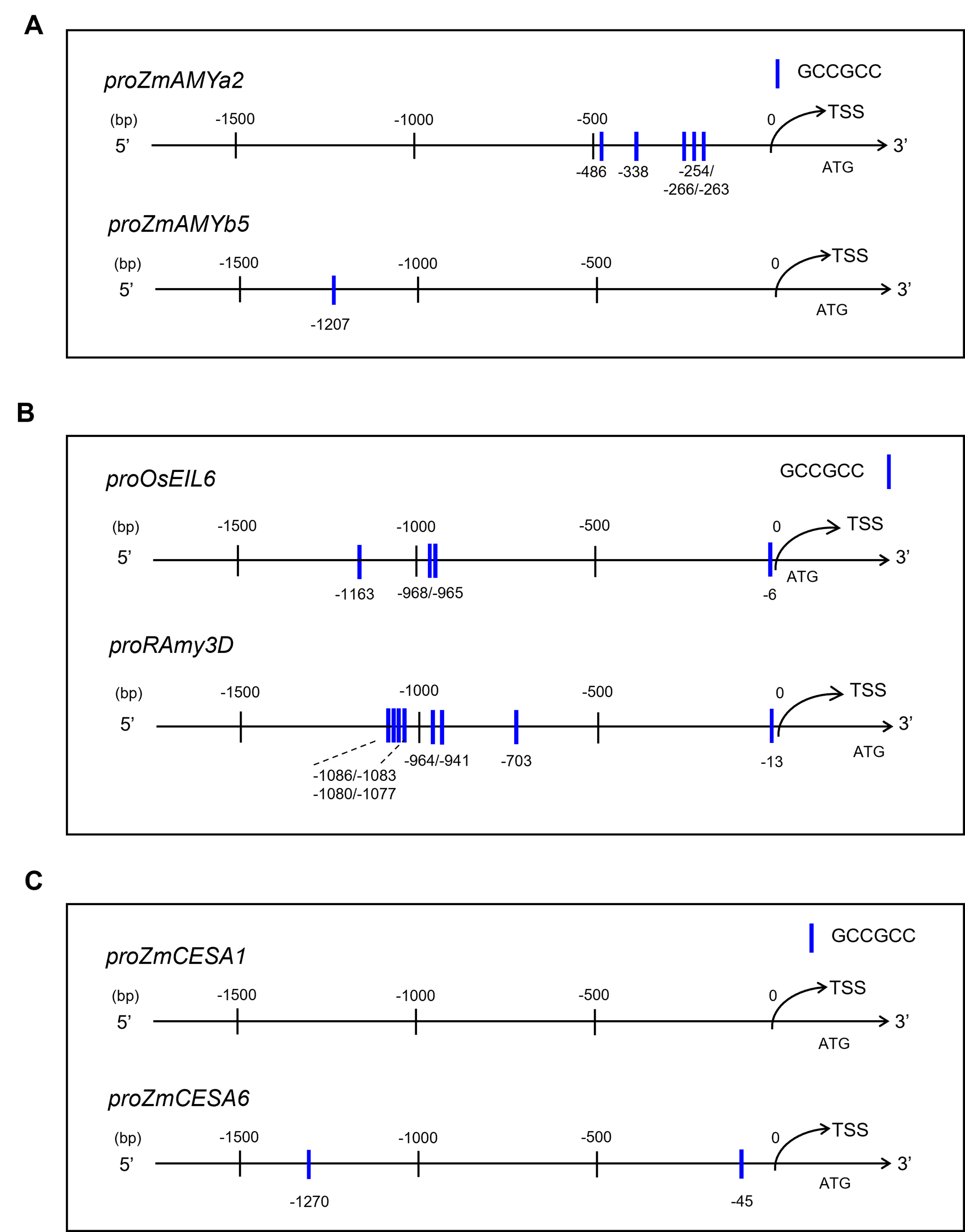

Supplement: S10 Fig — A-C. The identification of GCC-boxes for the promoters of ZmAMYs (A), OsEIL6 and RAmy3D (B) and ZmCESAs (C). The GCC-box was indicated with blue sticks. TSS, Transcription start site. (TIFF) [file pgen.1011052.s010.tiff]

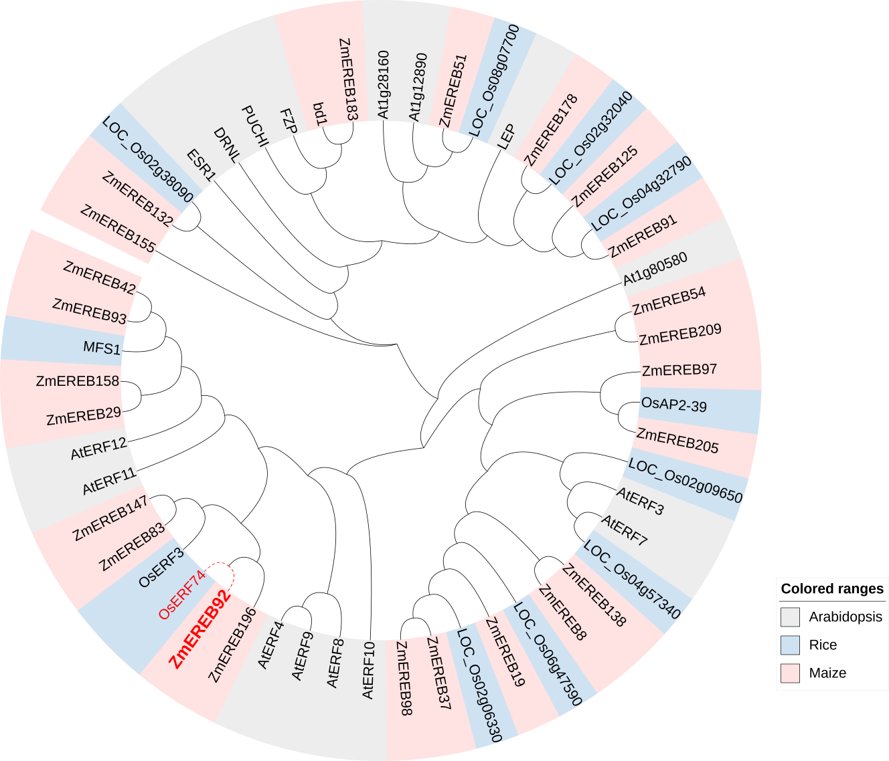

Supplement: S11 Fig — Phylogenetic analysis of ZmEREB92 with other group VIII ERF family members in maize, rice and Arabidopsis. MEGA software was used to perform the sequence alignment with ClustalW method and the phylogenetic trees was constructed with the Maximum-Likelihood method. The iTOL online website (https://itol.embl.de/) was used to visualize the tree. (TIF) [file pgen.1011052.s011.tif]

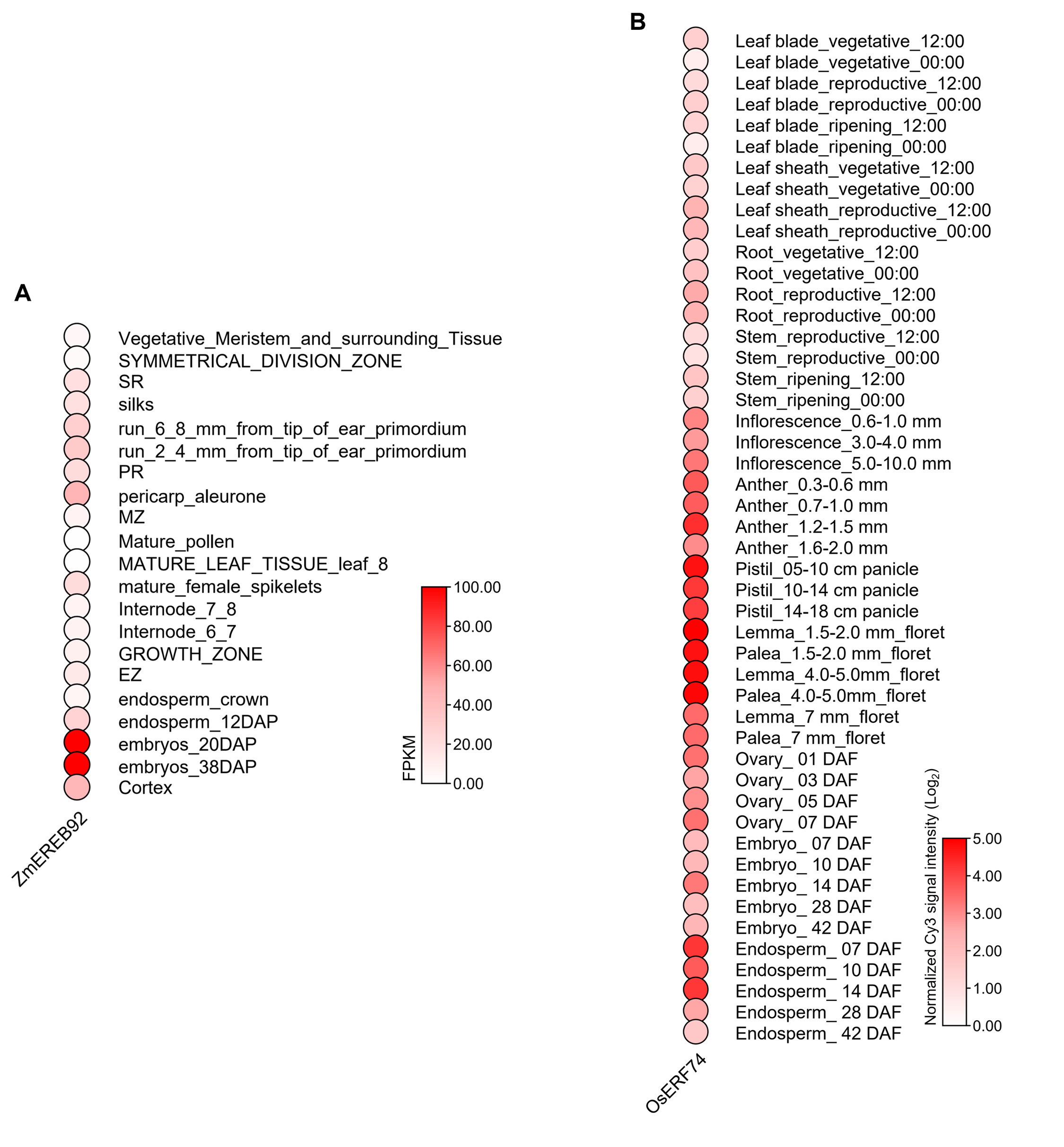

Supplement: S12 Fig — A-B. The heatmap showing expression profile of ZmEREB92 (A) and OsERF74 (B). (TIF) [file pgen.1011052.s012.tif]

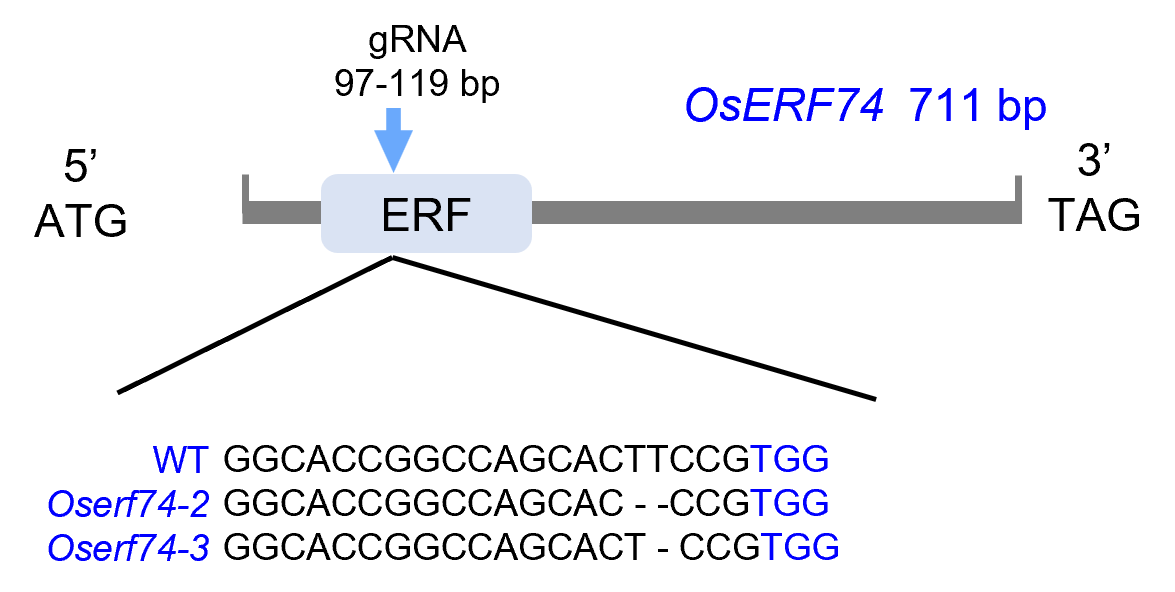

Supplement: S13 Fig — (TIF) [file pgen.1011052.s013.tif]

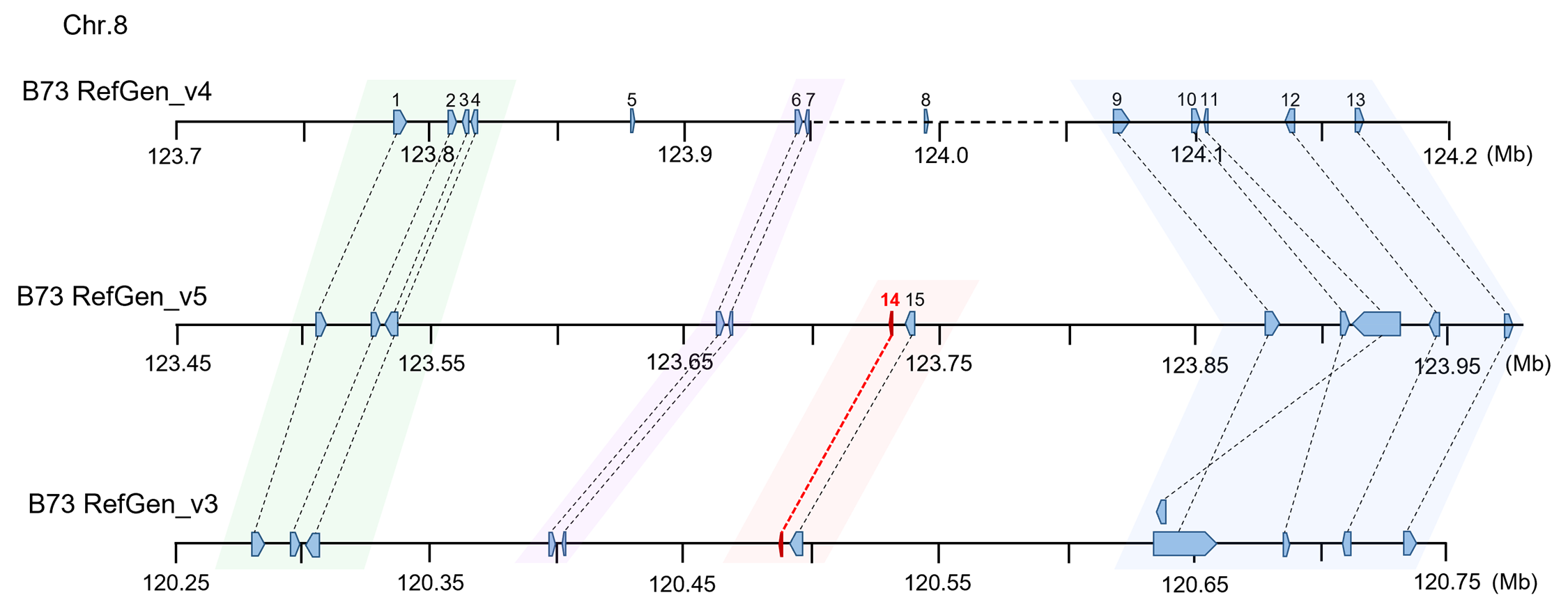

Supplement: S14 Fig — Comparison of 0.5 Mb genomic region across ZmEREB92 locus at chromosome 8 between three versions of B73 reference genome. The same genes are connected by dash lines. The numbers reflect different genes. ZmEREB92 is indicated by number 14 with red dash line. Detail information for genes were listed in S4 Table. (TIF) [file pgen.1011052.s014.tif]

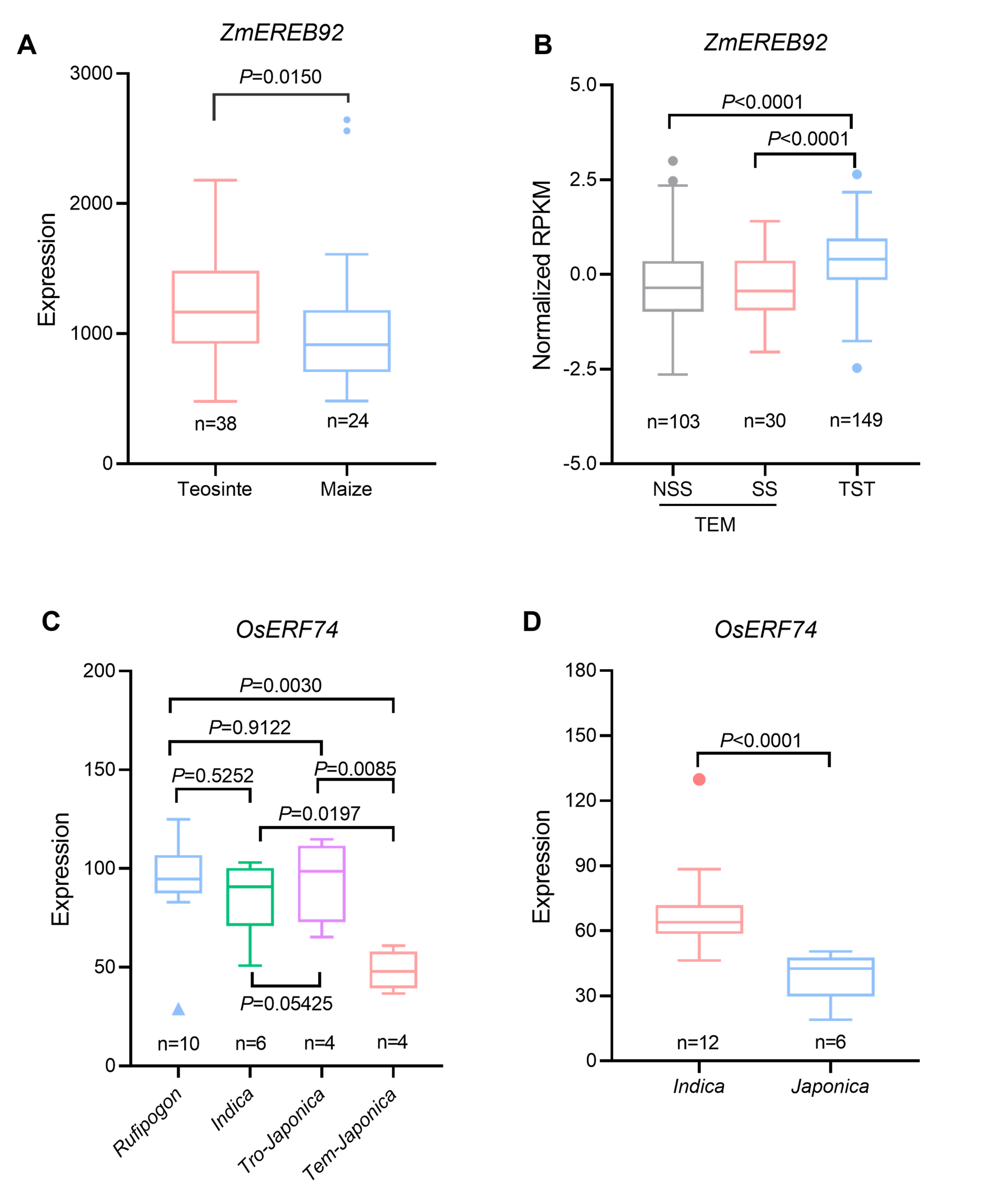

Supplement: S15 Fig — A. The expression level of ZmEREB92 in teosinte and cultivated maize. B. The normalized RPKM of ZmEREB92 in different maize subpopulations. NSS, non-stiff stalk. SS, stiff-stalk. TEM, temperate. TST, tropical/subtropical. C. The expression level of OsERF74 in wild and cultivated rice. Tro, tropical. Tem, temperate. D. The expression level of OsERF74 in different rice subspecies. A-D. The values were displayed Tukey box-plot. Number marked for each data is the exact P value (Mann-Whiney U test). (TIFF) [file pgen.1011052.s015.tiff]

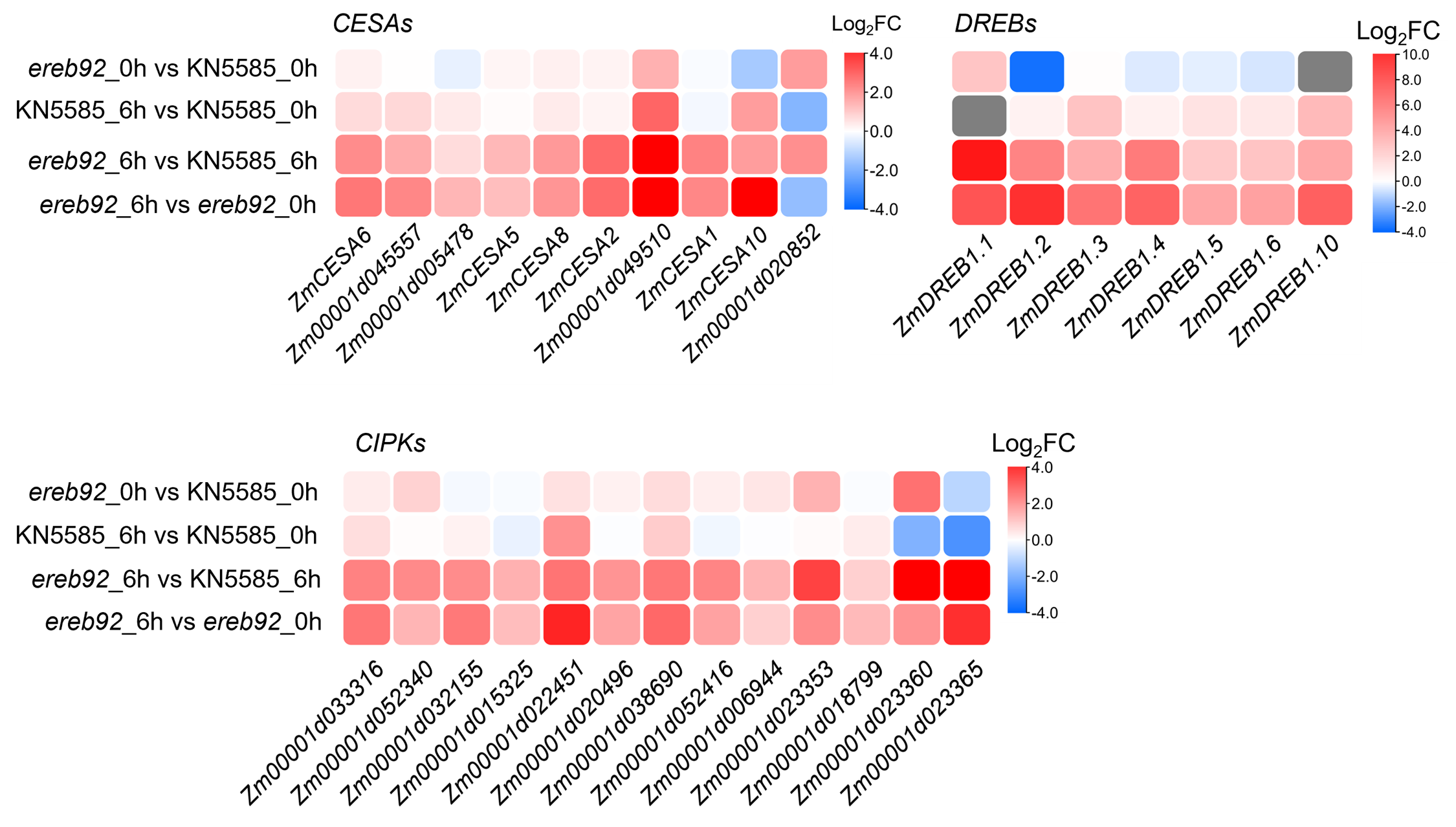

Supplement: S16 Fig — The heatmap shows the log2FC of differentially expressed DREBs and CIPKs in the comparison groups of ere92_6h vs ere92_0h or ere92_6h vs KN5585_6h. (TIF) [file pgen.1011052.s016.tif]

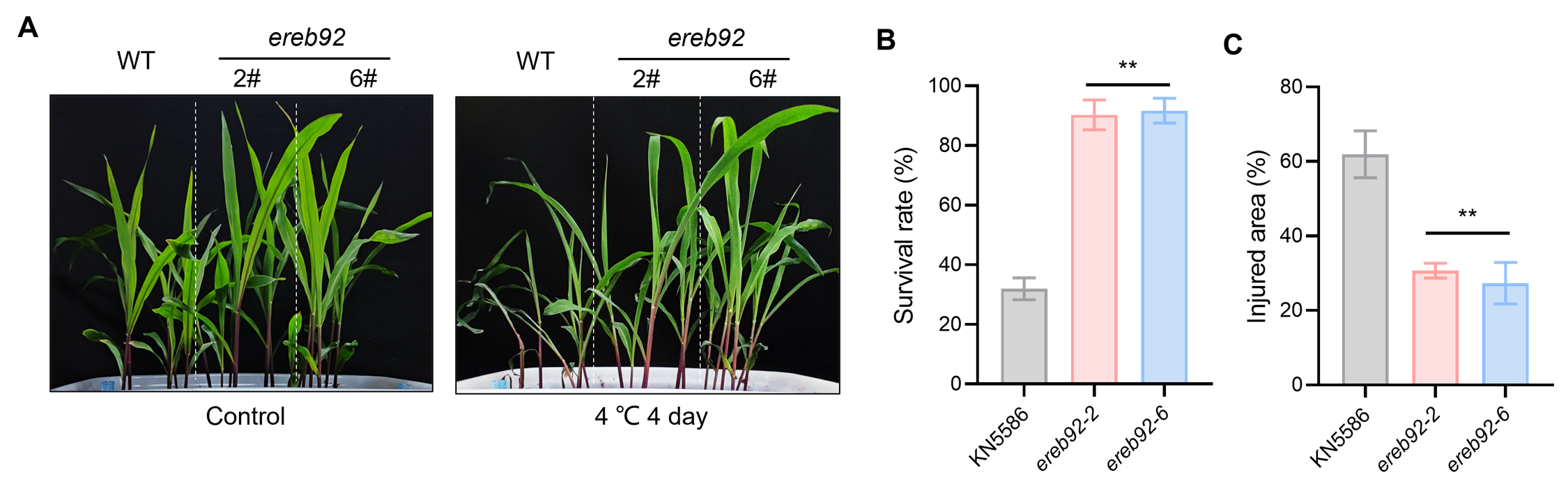

Supplement: S17 Fig — A-C. Chilling phenotype (A), injured area (B) and survival rate (C) of KN5585 and ereb92 mutants after 4 days treatment under cold condition (4 °C). (G, H) Error bars indicate mean ± SE (n = 6 for G, n = 3 for H). Asterisks indicate significant difference (Student’s t-test, **P<0.01). (TIF) [file pgen.1011052.s017.tif]

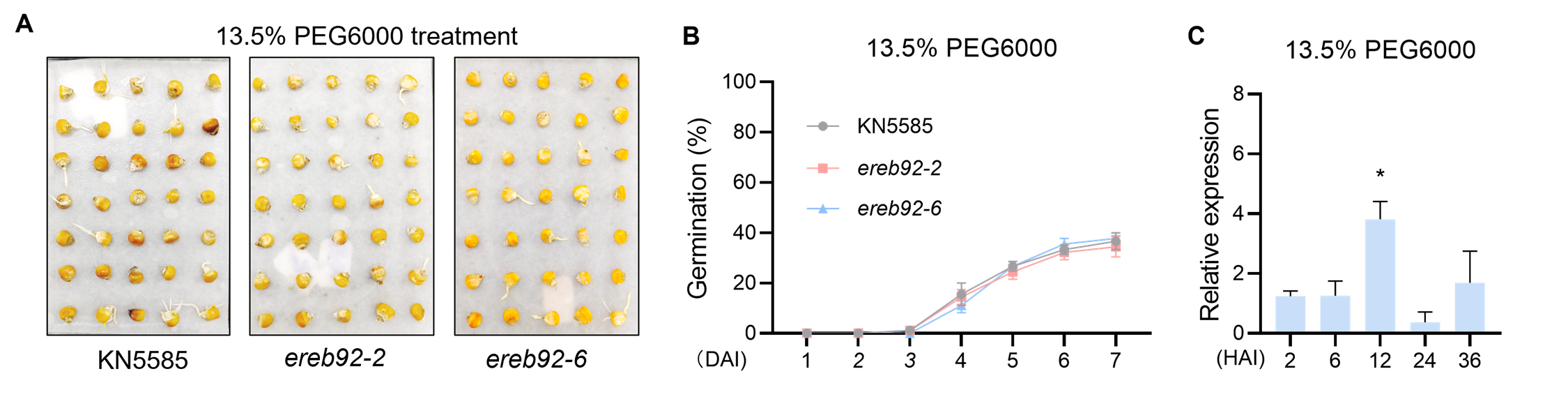

Supplement: S18 Fig — A. The germination performance of KN5585 and ereb92 mutants under 13.5% PEG6000 treatment at 5 DAI. B. The time course germination from 1–7 DAI of KN5585 and ereb92 mutants under 13.5% PEG6000 treatment. Error bars indicate mean ± SE (n = 3). The expression of ZmEREB92 in Mo17 seeds under 13.5% PEG6000 treatment at 2, 6, 12, 24 and 36 HAI. Error bars indicate mean ± SE (n = 3). Ef1a was used as the reference gene and relative expression level was normalized to one biological replicate of 2 HAI. Asterisks indicate significant difference compared to 2 HAI (one-way ANOVA followed by LSD tests, *P<0.05). (TIFF) [file pgen.1011052.s018.tiff]
